# METTL3 promotes tumour development by decreasing APC expression mediated by *APC* mRNA *N*^6^-methyladenosine-dependent YTHDF binding

**DOI:** 10.1038/s41467-021-23501-5

**Published:** 2021-06-21

**Authors:** Wei Wang, Fei Shao, Xueying Yang, Juhong Wang, Rongxuan Zhu, Yannan Yang, Gaoxiang Zhao, Dong Guo, Yingli Sun, Jie Wang, Qi Xue, Shugeng Gao, Yibo Gao, Jie He, Zhimin Lu

**Affiliations:** 1 Department of Thoracic Surgery, National Cancer Center/National Clinical Research Center for Cancer/Cancer Hospital, Chinese Academy of Medical Sciences and Peking Union Medical College, Beijing, China; 2 Department of Medical Oncology, National Cancer Center/National Clinical Research Center for Cancer/Cancer Hospital, Chinese Academy of Medical Sciences and Peking Union Medical College, Beijing, China; 3 Affiliated Hospital of Qingdao University and Qingdao Cancer Institute, Qingdao, Shandong China; 4 Zhejiang Provincial Key Laboratory of Pancreatic Disease, The First Affiliated Hospital and Institute of Translational Medicine, Zhejiang University School of Medicine, Hangzhou, China; 5 National Cancer Center/National Clinical Research Center for Cancer/Cancer Hospital and Shenzhen Hospital, Chinese Academy of Medical Sciences and Peking Union Medical College, Shenzhen, China; 6 State Key Laboratory of Molecular Oncology, National Cancer Center/National Clinical Research Center for Cancer/Cancer Hospital, Chinese Academy of Medical Sciences and Peking Union Medical College, Beijing, China; 7 Zhejiang University Cancer Center, Hangzhou, China

**Keywords:** Cancer, Cell biology, Molecular biology, Gastroenterology, Medical research

## Abstract

The adenomatous polyposis coli (*APC*) is a frequently mutated tumour suppressor gene in cancers. However, whether *APC* is regulated at the epitranscriptomic level remains elusive. In this study, we analysed TCGA data and separated 200 paired oesophageal squamous cell carcinoma (ESCC) specimens and their adjacent normal tissues and demonstrated that methyltransferase-like 3 (METTL3) is highly expressed in tumour tissues. m^6^A-RNA immunoprecipitation sequencing revealed that METTL3 upregulates the m^6^A modification of *APC*, which recruits YTHDF for *APC* mRNA degradation. Reduced APC expression increases the expression of β-catenin and β-catenin-mediated cyclin D1, c-Myc, and PKM2 expression, thereby leading to enhanced aerobic glycolysis, ESCC cell proliferation, and tumour formation in mice. In addition, downregulated APC expression correlates with upregulated METTL3 expression in human ESCC specimens and poor prognosis in ESCC patients. Our findings reveal a mechanism by which the Wnt/β-catenin pathway is upregulated in ESCC via METTL3/YTHDF-coupled epitranscriptomal downregulation of *APC*.

## Abstract (web summary)

The epitranscriptomic regulation of adenomatous polyposis coli (APC) tumour suppressor gene in cancers is unclear. Here the authors show that N6-methyladenosine methylation writer METTL3 downregulates APC by recruiting YTHDF2 for APC mRNA degradation, and that this promotes glycolysis and tumour growth in oesophageal cancers.

## Introduction

The adenomatous polyposis coli (*APC*) is known as a key tumour suppressor gene. APC plays a crucial role in suppressing the canonical Wnt signalling pathway, which controls cell proliferation and differentiation^1–3^. Loss of APC function leads to abnormal stabilization of β-catenin. Transactivated β-catenin forms a complex with T-cell factor/lymphoid enhancer-binding factor family members and induces many instrumental downstream genes, such as *CCND1* and *MYC*, which promote tumour development^4,5^. Mutation rates of *APC* in colorectal cancer can reach 80%^6–8^. In contrast, *APC* is rarely mutated in other types of cancer, such as oesophageal squamous cell carcinoma (ESCC), which has only ~1.5% *APC* mutation^9,10^. Whether *APC* expression is regulated at the epitranscriptomic level, thereby contributing to tumour development, has not been determined.

RNA modifications reveal a new level of posttranscriptional gene expression regulation^11–15^. *N*^6^-methyladenosine (m^6^A) modification is the most abundant RNA modification in eukaryotic mRNAs and is mainly mediated by m^6^A regulators, including ‘writers’, ‘erasers’ and ‘readers’, which add, remove and recognize methylation, respectively. m^6^A modification regulates RNA stability and translation efficiency, chromatin state, alternative polyadenylation and pre-mRNA splicing^16–20^. The identified writers include methyltransferase-like (METTL) 3/14, Wilms tumour 1-associated protein (WTAP), RNA-binding motif protein 15/15B and KIAA1429. Fat mass and obesity-associated protein (FTO) and alkB homologue 5 (ALKBH5) are erasers, whereas YT521-B homology (YTH) domain-containing proteins (YTH Domain Family [YTHDF] 1–3 and YTH Domain-Containing 1–2), heterogeneous nuclear ribonucleoprotein protein families and insulin growth factor-2 mRNA-binding proteins families are regarded as readers^21,22^. RNA m^6^A modification and its key m^6^A methyltransferase METTL3, which forms a heterodimer with METTL14, have been reported to be essential for tumour progression in several types of cancer^23–25^. However, the mechanism governing METTL3-promoted tumour progression has not been elucidated.

In this study, we demonstrate that METTL3 is upregulated in ESCC and enhances m^6^A of *APC* mRNA, thereby leading to recruitment of YTHDF for *APC* mRNA degradation and subsequent enhanced aerobic glycolysis, ESCC cell proliferation, tumour formation in mice and poor prognosis in patients.

## Results

### Upregulated METTL3 is correlated with poor survival in ESCC patients

**Fig. 1:**
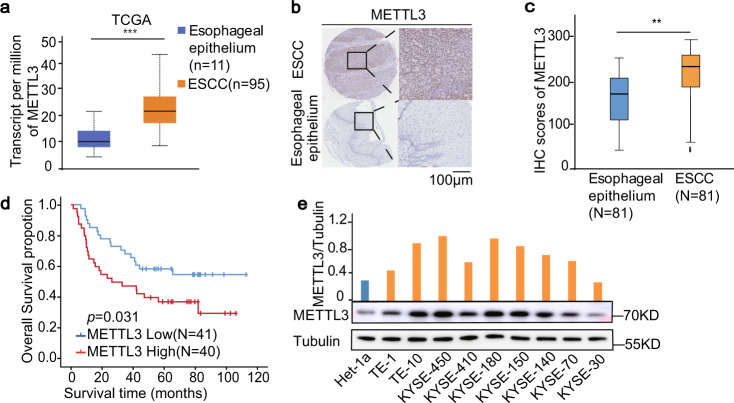
Upregulated METTL3 is correlated with poor survival in ESCC patients. **a** The expression levels (transcript per million) of *METTL3* were analysed in ESCC tissues (*n* = 95) and their adjacent normal tissues (*n* = 11) in the TCGA cohort. Data represent the means ± SD of samples. ***p = 2.21E − 07 based on two-tailed Student’s *t*-test. The box boundaries correspond to first and third quartiles; whiskers extend to a maximum of 1.5× the interquartile range. **b** Representative images of IHC staining for METTL3 protein on a tissue microarray (TMA) composed of 81 ESCC tissues and their adjacent normal epithelium tissues. scale bars: 100 μm. **c** The protein expression level of METTL3 were analysed in 81 ESCC tissues and their paired normal epithelium tissues by IHC. **p = 0.0013. Two-tailed *t*-test in paired samples. The box boundaries correspond to the first and third quartiles; whiskers extend to a maximum of 1.5× the interquartile range. **d** Kaplan–Meier method with two-tailed log-rank test was used to plot survival curves in human ESCC specimens (*n* = 81) with high and low METTL3 expression. The log-rank test was used to compare survival rate. **e** Immunoblotting analyses of nine ESCC cell lines and Het-1-immortalized normal cells were performed with the indicated antibodies. METTL3 expression levels were quantified and normalized to tubulin expression levels. Source data are provided as a Source Data file.

Oesophageal cancer is the sixth most common tumour in the world, with only a 12–20% 5-year patient survival rate being reported^26,27^. To explore the expression profile of METTL3 in ESCC, which constitutes ~90% of oesophageal cancer^28^, we analysed the Cancer Genome Atlas (TCGA) data and found that METTL3 expression was significantly upregulated in 95 ESCC specimens compared to that in 11 adjacent normal oesophageal epithelium tissues (Fig. 1a and Supplementary Fig. 1a). In addition, among the direct regulators of m^6^A, including METTL3, METTL14, WTAP, FTO and ALKBH5, we found that only METTL3 was consistently upregulated in ECSS tissues compared to normal tissues basing on the analyses of TCGA (Supplementary Fig. 1a) and GSE53625 (Supplementary Fig. 1b) dataset. Consistent with this result, microarray analysis of the gene expression profiles of 119 paired ESCC specimens and adjacent normal oesophageal epithelium tissues (Supplementary Fig. 1c), and immunohistochemical (IHC) staining of tissue arrays containing 81 paired ESCC specimens and adjacent normal oesophageal epithelium tissues (Fig. 1b, c) showed that METTL3 expression levels were significantly higher in the ESCC tissues than in the paired adjacent normal tissues. Kaplan–Meier analysis showed that the patients with high *METTL3* expression had shorter overall survival time than those with low *METTL3* expression (Fig. 1d and Supplementary Fig. 1d). In line with this finding, METTL3 mRNA (Supplementary Fig. 1e) and protein (Fig. 1e) expression levels were higher in nine different ESCC cell lines than in Het-1a-immortalized normal oesophageal epithelial cells. These results indicate that METTL3 expression is upregulated in ESCC and inversely correlated with ESCC patient survival time.

### METTL3 promotes ESCC cell proliferation and tumour growth in mice

**Fig. 2:**
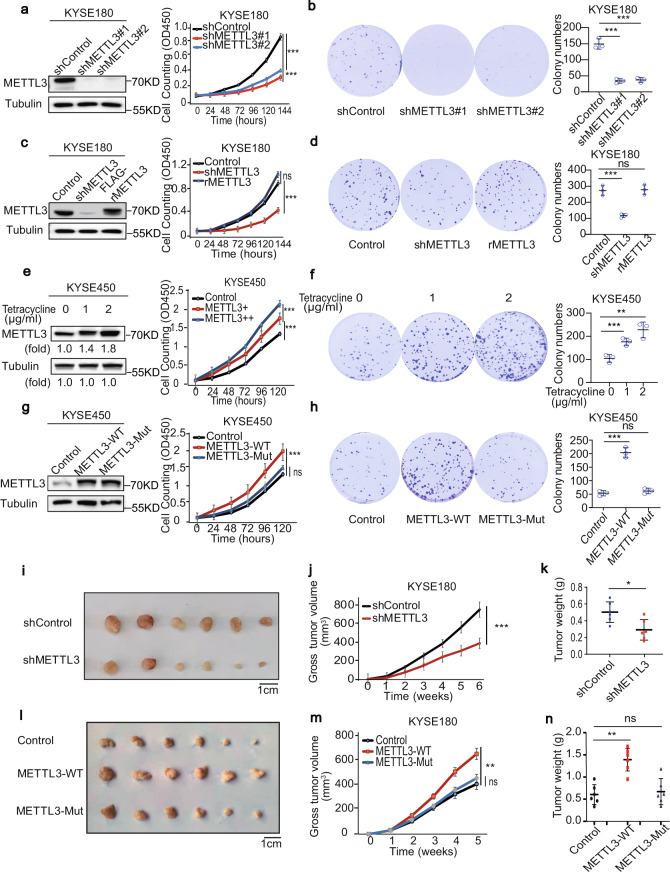
METTL3 promotes ESCC cell proliferation and tumour growth in mice. **a**–**d** KYSE180 cells with or without METTL3 depletion (**a**), or with reconstituted expression of an RNAi-resistant (r) METTL3 (**c**) were analysed by immunoblotting analyses. The cell proliferation (**a**, **c**) and colony formation (**b**, **d**) assays were performed. Data represent means ± SD of triplicate samples. **a** ***p = 0.0004 (top), 0.0002 (bottom). **b** ***p = 0. 0004 (left), 0.0004 (right) (two-tailed *t*-test). **c** ***p = 4.72E − 05. **d** ***p = 0.0007. Two-tailed *t*-test; ns, not significant. **e**, **f** KYSE450 cells expressing the METTL3 expression-inducible Tet-on lentiviral vector were treated with or without the indicated dosages of tetracycline, followed by immunoblotting analyses. The cell proliferation (**e**) and colony formation (**f**) assays were performed. Data represent means ± SD of triplicate samples. **e** ***p = 6.37E − 04 (top), ***p = 9.58E − 04 (bottom). **f** ***p = 9.24E − 04 (left), **p = 0.0095 (right). Two-tailed *t*-test. **g**, **h** KYSE450 cells with or without expressing WT METTL3 or an inactive METTL3 mutant were analysed by immunoblotting analyses. The cell proliferation (**g**) and colony formation (**h**) assays were performed. Data represent means ± SD of triplicate samples. ***p = 0.0004 (**g**), ***p = 0.0003 (**h**). Two-tailed *t*-test; ns, not significant. **i**–**k** KYSE180 cells with or without METTL3 depletion were subcutaneously injected into the mice (*n* = 6). Six weeks later, tumour sizes (**i**), volumes (**j**) and weight (**k**) were measured. Scale bar, 1 cm. Data represent means ± SD of six mice per group. ***p = 2.58E − 06 (**j**), *p = 0.0138 (**k**) (two-tailed *t*-test). **l**–**n** KYSE180 cells with or without expressing WT METTL3 or an inactive METTL3 mutant were subcutaneously injected into the mice (*n* = 6). Five weeks later, tumour sizes (**l**), volumes (**m**) and weight (**n**) were measured. Scale bar, 1 cm. Data represent means ± SD of six mice per group. **p = 0.0015 (**m**), 0.0013 (**n**) (two-tailed *t*-test). ns, not significant. Source data are provided as a Source Data file.

To determine the role of METTL3 in cell proliferation, we depleted METTL3 in KYSE180 (Fig. 2a) and KYSE450 (Supplementary Fig. 2a) ESCC cells by expressing two different *METTL3* short hairpin RNAs (shRNAs). We showed that METTL3 depletion reduced the proliferation (Fig. 2a and Supplementary Fig. 2a) and colony numbers (Fig. 2b and Supplementary Fig. 2b) of these cells. These inhibitory effects were abrogated by reconstituted expression of an RNA interference-resistant (r) Flag-tagged METTL3 (Flag-rMETTL3) in KYSE180 (Fig. 2c, d) and KYSE450 (Supplementary Fig. 2c, d).

We next established tetracycline-inducible expression of METTL3 in KYSE450 cells. Tetracycline treatment increased METTL3 expression in a dosage-dependent manner, which elicited correspondingly increased levels of cell proliferation (Fig. 2e) and colony formation (Fig. 2f). Compared to overexpression of wild-type (WT) METTL3, overexpression of a METTL3-inactive mutant in ESCC cells failed to largely promote cell growth and proliferation (Fig. 2g, h and Supplementary Fig. 2e, f). These results strongly suggested that METTL3 promotes tumour cell proliferation dependent on its expression levels and intact activity.

To determine the role of METTL3 in tumour growth in mice, we subcutaneously injected KYSE180 or KYSE450 cells with or without METTL3 depletion or overexpression of WT METTL3 or the METTL3-inactive mutant into athymic nude mice. We showed that METTL3 depletion reduced tumour size (Fig. 2i and Supplementary Fig. 2g), volume (Fig. 2j and Supplementary Fig. 2h) and weight (Fig. 2k and Supplementary Fig. 2i). Compared to the limited effect elicited by expression of the METTL3-inactive mutant, WT METTL3 overexpression greatly promoted tumour growth (Fig. 2l–n and Supplementary Fig. 2j–l). These results indicate that METTL3 expression is instrumental for tumour growth in mice.

### METTL3 mediates m^6^A upregulation on APC mRNA

**Fig. 3:**
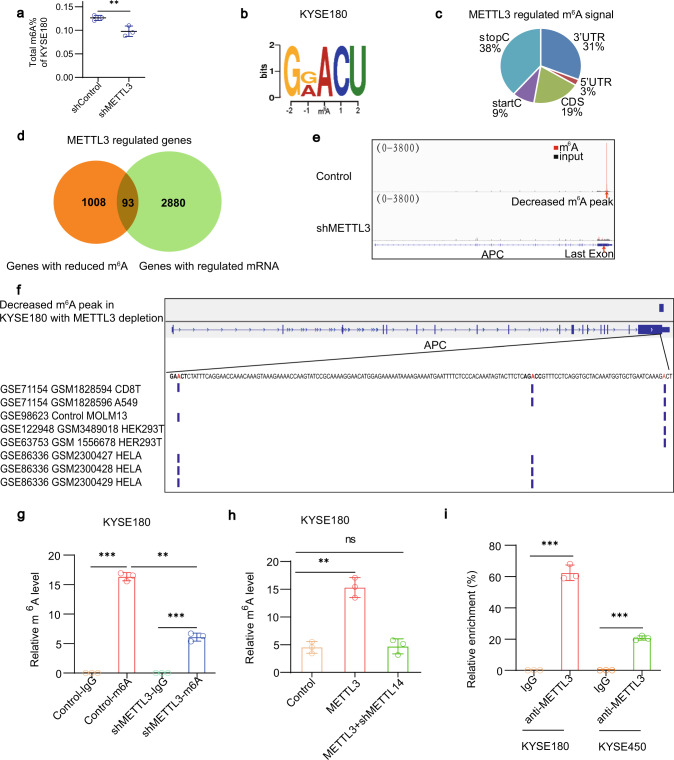
METTL3-mediated m^6^A upregulation on *APC* mRNA. **a** m^6^A content of total RNA in KYSE180 cells with or without METTL3 depletion was determined. Data represent the means ± SD of triplicate samples. **p = 0.0094 based on two-tailed Student’s *t*-test. **b** The most frequent m^6^A motifs detected by DREME software in MeRIP-seq of KYSE180 cells are shown. **c** MeRIP-seq of KYSE180 cells with or without METTL3 depletion was conducted. The proportions of m^6^A peak distribution in the 5′-untranslated region (5′-UTR), start codon, coding region (CDS), stop codon and the 3′-untranslated region (3′-UTR) across the entire set of mRNA transcripts were calculated. **d** The genes with diminished m^6^A peaks identified by MeRIP-seq (1101 genes, fold-change > 2) and the genes with upregulated or downregulated mRNA expression (2973 genes, fold-change > 2) identified by RNA-seq in KYSE180 with or without METTL3 depletion were analysed (93 genes overlapped). **e** The m^6^A peak visualization of m^6^A-seq in *APC* transcripts in KYSE180 cells with or without METTL3 depletion is shown. The m^6^A peaks are in the last exon of *APC*. **f** The MeRIP-Seq peak in KYSE180 regulated by METTL3 depletion and miCLIP peaks in other types of cancer cells from the Gene Expression Omnibus were aligned. The three methylated adenosine bases were denoted with red colour. **g**, **h** Methylated RNA in KYSE180 cells with or without METTL3 depletion (**g**), METTL3 expression or METTL3 and METTL14 shRNA expression (**h**) was immunoprecipitated with an m^6^A antibody followed by qPCR analyses with primers against *APC* mRNA. Data represent means ± SD of triplicate samples. **g** ***p = 3.84E − 05 (left), 8.99E − 05 (right); **p = 0.0012 (middle). **h** **p = 0.0019. Two-tailed *t*-test; ns, not significant. **i** RIP analyses of KYSE180 and KYSE450 cells were performed with an anti-METTL3 antibody followed by qPCR analyses with primers against *APC* mRNA. Data represent means ± SD of triplicate samples. ***p = 2.50E − 05 (left), 1.08E − 05 (right). Two-tailed *t*-test. Source data are provided as a Source Data file.

To determine the mechanism underlying METTL3-promoted tumour cell proliferation, we examined the role of METTL3 in epitranscriptomal regulation in ESCC cells. METTL3 depletion reduced the total m^6^A abundance in KYSE180 cells (Fig. 3a). Methylated RNA immunoprecipitation (MeRIP) with an m^6^A-specific antibody followed by RNA sequencing (MeRIP-seq) revealed that the identified m^6^A sites in the mRNAs of KYSE180 are consistent with the m^6^A consensus sequence RRACH (R = G or A; H = A, C or U)^15^, especially GRACU (*p* = 1.5e10 − 31) (Fig. 3b). In line with previous reports^15^, the m^6^A signal was enriched around the stop codon and 3′-untranslated region (UTR) of mRNAs (Fig. 3c).

METTL3 depletion reduced 1199 m^6^A peaks in mRNAs of 1101 genes (fold-change > 2) in MeRIP-seq, which included the transcripts that were well known to be modified by m^6^A, such as *ACTB*, *EEF2* and *MALAT1* long non-coding RNA (Supplementary Fig. 3a)^29^, and regulated expression of 2973 genes (fold-change > 2) in mRNA sequencing (Supplementary Data 1). Among these genes, 93, including *APC*, were overlapped (Fig. 3d). Gene Ontology (GO) enrichment analyses of cellular component (CC) terms in MeRIP-seq showed that m^6^A levels were substantially decreased in the gene set of the β-catenin destruction complex (Supplementary Fig. 3b), which included *APC*, in KYSE180 cells with METTL3 depletion.

APC is a negative Wnt regulator that promotes β-catenin degradation^1–3^. We showed that the m^6^A peak is in the last exon near the termination codon region of *APC*, and that METTL3 depletion reduced the m^6^A level of *APC* mRNA (Fig. 3e). These results are in line with previous reports showing that m^6^A level of *APC* mRNA were high in different types of cancer cells, such as HeLa cervical cancer cells, H1299 lung cancer cells and HepG2 liver cancer cells, within the top 10% of most methylated transcripts (Supplementary Fig. 3c)^17,30–36^. Analyses of available m^6^A individual-nucleotide-resolution cross-linking and immunoprecipitation (miCLIP) data^37–41^ revealed that three adenosine bases were methylated and are within the decreased m^6^A peak of *APC* mRNA by METTL3 depletion (Fig. 3f). These results indicate that METTL3 regulates the m^6^A level of *APC* mRNA, which is high in many types of cancer cells.

m^6^A RIP followed by real-time quantitative PCR assay showed that m^6^A levels in *APC* mRNA were notably decreased upon METTL3 depletion in KYSE180 (Fig. 3g) and KYSE450 (Supplementary Fig. 3d) cells. Conversely, METTL3 overexpression increased m^6^A levels in *APC* mRNA in KYSE180 (Fig. 3h) and KYSE450 (Supplementary Fig. 3e) cells, and this increase was abrogated by METTL14 depletion (Fig. 3h and Supplementary Fig. 3e). In addition, RIP with an anti-METTL3 antibody showed that METTL3 bound to *APC* mRNA in KYSE180 and KYSE450 cells (Fig. 3i). These results indicate that METTL3 binds to *APC* mRNA and enhances its m^6^A levels in an METTL14-dependent manner.

### METTL3-dependent m^6^A upregulation on *APC* mRNA suppresses APC expression

**Fig. 4:**
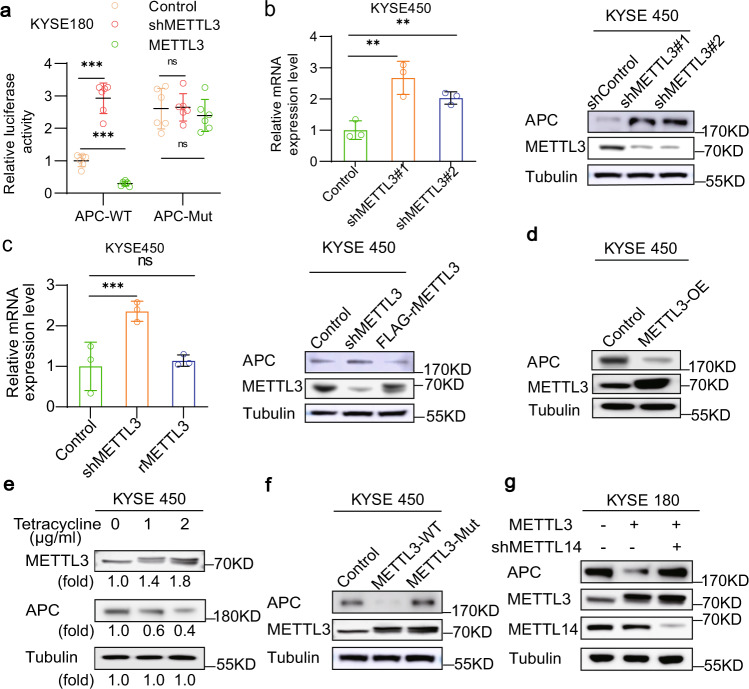
METTL3-dependent m^6^A upregulation on *APC* mRNA suppresses APC expression. **a** Luciferase vectors with the WT or mutated m^6^A nucleotides in the *APC* gene were transfected into KYSE180 cells with or without METTL3 depletion or METTL3 overexpression. Relative luciferase activity was measured. Data represent means ± SD of triplicate samples. ***p = 2.93E − 06 (left), 5.88E − 06 (right). Two-tailed *t*-test; ns, not significant. **b**, **c** The relative mRNA and protein expression levels of *APC* in KYSE450 cells with or without METTL3 depletion (**b**), or with reconstituted expression of Flag-rMETTL3 (**c**) were determined by qPCR and immunoblotting analyses with the indicated antibodies, respectively. Data represent means ± SD of triplicate samples. **b** **p = 0.0088 (left), 0.0074 (right). **c** ***p = 0.0004. Two-tailed *t*-test; ns, not significant. **d**–**g** KYSE450 cells were transfected with or without a plasmid expressing METTL3 (**d**). KYSE450 cells expressing the METTL3 expression-inducible Tet-on lentiviral vector were treated with or without the indicated dosages of tetracycline **e**. KYSE450 cells were transfected with or without plasmids expressing WT METTL3, an inactive METTL3 mutant (**f**), WT METTL3 or METTL3 and METTL14 shRNA (**g**). Immunoblotting analyses were performed for three times with similar results. Source data are provided as a Source Data file.

To determine the effect of METTL3-dependent m^6^A regulation on *APC* expression, we constructed a luciferase reporter gene with an integrated coding sequence (CDS) containing the WT or mutated m^6^A from the 3′-end of *APC* mRNA, in which the three adenosine bases within the m^6^A consensus sequences were mutated to thymine (T) (Supplementary Fig. 4a). Luciferase assays showed that METTL3 depletion largely increased the activity of luciferase with the WT *AP*C, whereas the mutated *AP*C elevated the luciferase activity and rendered this activity resistant to regulation by METTL3 depletion (Fig. 4a). In contrast, METTL3 overexpression suppressed the activity of luciferase with WT *AP*C but not mutated *AP*C (Fig. 4a). These results suggest that METTL3-mediated m^6^A level upregulation of *APC* mRNA suppresses APC expression.

Consistent with this finding, METTL3 depletion increased *APC* mRNA and protein expression (Fig. 4b and Supplementary Fig. 4b), and these increases were abrogated by reconstituted expression of rMETTL3 in both KYSE450 (Fig. 4c) and KYSE180 (Supplementary Fig. 4c) cells. In addition, overexpression of METTL3 decreased APC expression in KYSE450 cells (Fig. 4d) and tetracycline dosage-dependently increased METTL3 expression levels were inversely correlated with APC levels (Fig. 4e). In contrast, METTL3-inactive mutant, unlike its WT counterpart, failed to modulate APC expression (Fig. 4f and Supplementary Fig. 4d). Of note, METTL14 depletion in the ESCC cells abrogated the METTL3 overexpression-decreased APC expression (Fig. 4g and Supplementary Fig. 4e). These results indicate that *APC* mRNA and protein expression are suppressed by *APC* mRNA m^6^A upregulation mediated by METTL3 in concert with METTL14 in ESCC cells.

### METTL3-enhanced m^6^A of *APC* mRNA and subsequent binding of YTHDF suppresses APC expression

**Fig. 5:**
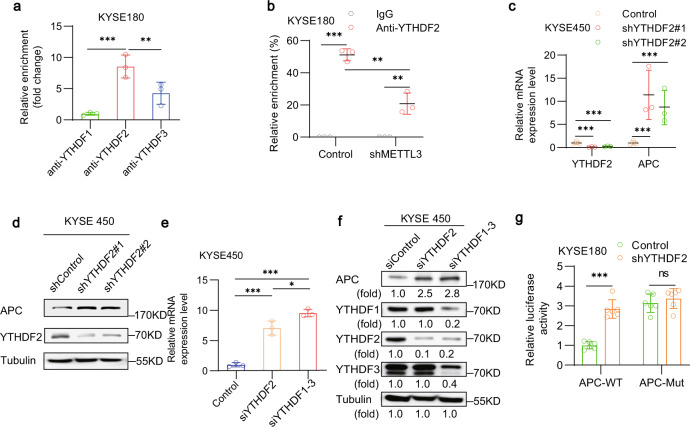
METTL3-enhanced m^6^A of *APC* mRNA and subsequent binding of YTHDF2 suppresses APC expression. **a** RIP analyses of KYSE180 cells were performed with an anti-YTHDF1 or anti-YTHDF2, or anti-YTHDF3 antibody followed by qPCR analyses with primers against *APC* mRNA. Data represent the means ± SD of triplicate samples. ***p = 0.0006 (left), **p = 0.0092 (right) based on two-tailed Student’s *t*-test. **b** KYSE180 cells were transfected with or without a vector expressing METTL3 shRNA. RIP analyses were performed with an anti-YTHDF2 antibody followed by qPCR analyses with primers against APC mRNA. Data represent the means ± SD of triplicate samples. ***p = 1.85E − 05 (left), **p = 0.0023 (middle) and 0.0056 (right) based on two-tailed Student’s *t*-test. **c** KYSE450 cells were transfected with or without a vector expressing YTHDF2 shRNA. The relative mRNA expression levels of *APC* were measured using quantitative PCR. Data represent the means ± SD of triplicate samples. ***p = 0.0006, 0.0009, 5.58E − 05 and 0.0007 (left to right) based on two-tailed Student’s *t*-test. **d** KYSE450 cells were transfected with or without YTHDF2 shRNA. Immunoblotting analyses were performed with the indicated antibodies for three times with similar results. **e** KYSE180 cells were transfected with or without a YTHDF2 siRNA or combination of YTHDF1–3 siRNAs. The relative mRNA expression levels of APC were measured using quantitative PCR. Data represent the means ± SD of triplicate samples. ***p = 9.33E − 04 (left) and 2.99E − 05 (right), *p = 0.031 based on two-tailed Student’s *t*-test. **f** KYSE180 cells were transfected with or without a YTHDF2 siRNA or combination of YTHDF1–3 siRNAs. Immunoblotting analyses were performed with the indicated antibodies for three times with similar results. **g** KYSE180 cells expressing luciferase reporter genes fused with or without the wild-type (WT) or mutated m6A nucleotides from *APC* genes were transfected with or without a vector expressing YTHDF2 shRNA. The relative luciferase activity after normalization to the shControl group is shown. Data represent the means ± SD of triplicate samples. ***p = 3.94E − 06 based on two-tailed Student’s *t*-test. ns, not significant. Source data are provided as a Source Data file.

m^6^A readers bind to m^6^A-modified mRNAs, leading to increased or decreased protein expression. YTHDF1–3 mediate mRNA degradation^30,42–45^. Analyses of the published YTHDF1–3 CLIP data sets showed that YTHDF1–3 bound *APC* mRNA (Supplementary Fig. 5a)^18,44,46,47^. RIP analyses with antibodies against YTHDF1, YTHDF2 or YTHDF3 followed by real-time quantitative PCR assay revealed that the amount of YTHDF2 that bound *APC* mRNA was much more than those of YTHDF1 and YTHDF3 in KYSE180 (Fig. 5a) and KYSE450 (Supplementary Fig. 5b), and was reduced by METTL3 depletion (Fig. 5b and Supplementary Fig. 5c). These results suggest that METTL3-increased m^6^A of *APC* mRNA largely promotes the binding of YTHDF2 to *APC* mRNA.

To examine the role of the binding of YTHDF2 to *APC* mRNA in *APC* expression, we depleted YTHDF2, which increased the mRNA (Fig. 5c and Supplementary Fig. 5d) and protein (Fig. 5d and Supplementary Fig. 5e) expression of *APC* in KYSE450 (Fig. 5c, d) and KYSE180 (Supplementary Fig. 3d, e) cells. Combined depletion of YTHDF1–3 further increased the mRNA (Fig. 5e and Supplementary Fig. 5f) and protein (Fig. 5f and Supplementary Fig. 5g) expression of *APC* in these cells. A similar increase was also observed in HeLa cells with depletion of YTHDF1–3 (Supplementary Fig. 5h). In addition, we performed a luciferase reporter assay and showed that depletion of YTHDF2 enhanced the activity of luciferase driven by the WT *APC* CDS sequence containing m^6^A, whereas the mutated *AP*C enhanced the luciferase activity and rendered this activity resistant to regulation by YTHDF2 depletion (Fig. 5g). In addition, METTL3 overexpression-suppressed luciferase activity driven by the WT *APC* CDS sequence was restored by YTHDF2 depletion, which did not affect the mutated *AP*C-enhanced luciferase activity (Supplementary Fig. 5i). These results indicate that METTL3-increased m^6^A of *APC* mRNA and subsequent binding of YTHDF suppress APC expression.

### METTL3 reduces APC expression and promotes β-catenin-mediated downstream gene expression, aerobic glycolysis, ESCC cell proliferation and tumour development

**Fig. 6:**
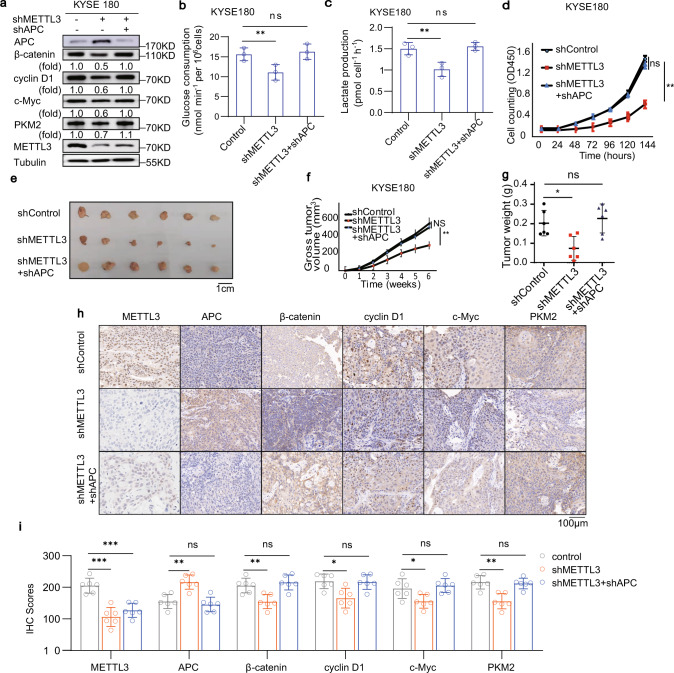
METTL3 reduces APC expression and promotes β-catenin downstream gene expression, aerobic glycolysis, ESCC cell proliferation and tumour development. **a** Immunoblotting analyses of KYSE180 cells with or without METTL3 shRNA expression or combined METTL3 shRNA and APC shRNA expression were performed with the indicated antibodies for three times with similar results. **b**, **c** Glucose consumption (**b**) and lactate production (**c**) of KYSE180 cells with or without METTL3 shRNA expression, or combined METTL3 shRNA and APC shRNA expression were determined. Data represent the means ± SD of triplicate samples. **p = 0.0093 (**b**) and *p* = 0.0062 (**c**) based on two-tailed Student’s *t*-test. ns, not significant. **d** KYSE180 cells with or without METTL3 shRNA expression or combined METTL3 shRNA and APC shRNA expression were cultured for the indicated periods of time and collected for cell counting. Data represent the means ± SD of triplicate samples. **p = 0.0023 based on two-tailed Student’s *t*-test. ns, not significant. **e**–**i** KYSE180 cells with or without METTL3 shRNA expression or combined METTL3 shRNA and APC shRNA expression were subcutaneously injected into the flank regions of nude mice (*n* = 6). Six weeks later, tumour sizes (**e**), volumes (**f**) and weight (**g**) were measured. Data represent the means ± SD of six mice in each group. **p = 0.0025 (**f**), *p = 0.0135 (**g**) based on two-tailed Student’s *t*-test. **h** IHC staining of tumour tissues was performed with the indicated antibodies for three times with similar results. Representative images are shown. Scale bar, 100 μm. **i** The protein expression levels of the indicated proteins were displayed. *p = 0.0269, **p = 0.0033, 0.0069 and 0.0011 (left to right), ***p = 8.35E – 05, 0.0002 and 0.0008 (left to right) based on two-tailed Student’s *t*-test. ns, not significant. Source data are provided as a Source Data file.

Loss of APC function stabilizes β-catenin to induce the expression of downstream genes, such as *CCND1* and *MYC*^4,48,49^. Cyclin D1 (encoded by *CCND1*) promotes cell cycle progression, whereas c-Myc (encoded by *MYC*) enhances aerobic glycolysis through upregulation of glycolytic genes, such as pyruvate kinase M2 isozyme (PKM2) expression^50–52^. As expected, METTL3 depletion enhanced APC expression and reduced expression of β-catenin, cyclin D1, c-Myc and PKM2 in KYSE180 (Fig. 6a) and KYSE450 (Supplementary Fig. 6a) cells. On the contrary, METTL3 overexpression reduced APC expression and enhanced expression of β-catenin, cyclin D1, c-Myc and PKM2 in KYSE180 (Supplementary Fig. 6b) and KYSE450 (Supplementary Fig. 6c) cells, and this METTL3 overexpression-induced effect was eliminated by METTL14 depletion (Supplementary Fig. 6b, c). In addition, METTL3 depletion decreased glucose uptake (Fig. 6b and Supplementary Fig. 6d), lactate production (Fig. 6c and Supplementary Fig. 6e), cell proliferation (Fig. 6d and Supplementary Fig. 6f) and numbers of cell colonies (Supplementary Fig. 6g). Notably, this METTL3 depletion-elicited inhibition of gene expression (Fig. 6a), glycolysis (Fig. 6b, c), cell proliferation (Supplementary Fig. 6f) and colony formation (Supplementary Fig. 6g) was largely abrogated by APC depletion in these cells. Conversely, METTL3 overexpression elicited an increase of glucose consumption and lactate production, which was abrogated by YTHDF2 depletion (Supplementary Fig. 6h–k). In addition, metabolic flux analysis of ^13^C_6_ glucose-labelled KYSE180 cell showed that METTL3 overexpression-enhanced glycolysis was reduced by c-Myc depletion (Supplementary Fig. 6l). These results indicate that METTL3 reduces APC expression and promotes β-catenin-mediated downstream gene expression, aerobic glycolysis and ESCC cell proliferation.

To determine the role of METTL3-induced APC expression downregulation in tumour growth in mice, we subcutaneously injected KYSE180 and KYSE450 cells with or without METTL3 depletion, or with or without combined METTL3 and APC depletion into athymic nude mice. We showed that METTL3 depletion reduced tumour size (Fig. 6e and Supplementary Fig. 7a), volume (Fig. 6f and Supplementary Fig. 7b) and weight (Fig. 6g and Supplementary Fig. 7c), as well as the amount of lactate in the tumour tissue (Supplementary Fig. 7d) were restored by APC depletion. In addition, IHC staining showed that METTL3 depletion increased APC expression with corresponding decreased expression of β-catenin, cyclin D1, c-Myc and PKM2 in tumour tissues (Fig. 6h, i). These METTL3 depletion-induced alteration of protein expression were abrogated by APC depletion. Consistent with the tumour-promoting effect of METTL3 overexpression (Fig. 2l–n), overexpression of WT, but not the inactive mutant, METTL3 increased the amount of lactate in tumour tissue (Supplementary Fig. 7e). These results indicate that suppression of APC expression by METTL3 promotes tumour development.

### Downregulated APC expression correlates with upregulated METTL3 expression in human ESCC specimens and poor prognosis of ESCC patients

**Fig. 7:**
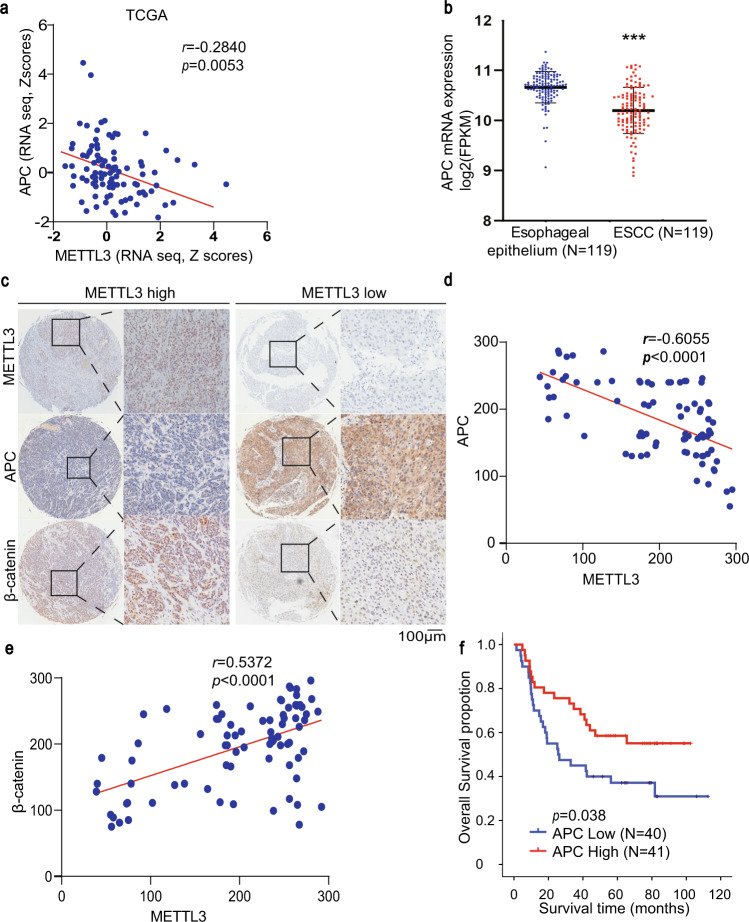
Downregulated APC expression correlates with upregulated METTL3 expression in human ESCC specimens and poor survival of ESCC patients. **a** The correlation between the mRNA levels of *METTL3* and *APC* in ESCC of TCGA was analysed by the two-tailed Pearson correlation coefficient. **b** The relative mRNA expression levels of *APC* were analysed in 119 paired ESCC tissues and their adjacent normal tissues (from the GSE53625 dataset). Data represent the means ± SD of 119 samples. ***p = 1.75E − 15 based on two-tailed and paired Student’s *t*-test. **c** IHC staining of 81 ESCC specimens was performed with the indicated antibodies three times with similar results. Representative images of METTL3, APC and β-catenin expression levels in two cases of ESCC are shown. Scale bar, 100 μm. **d** The correlation between the expression levels of METTL3 and APC in 81 ESCC specimens was analysed by the two-tailed Pearson correlation coefficient. *p* < 0.0001. **e** The correlation between the expression levels of METTL3 and β-catenin in 81 ESCC specimens was analysed by the two-tailed Pearson correlation coefficient. *p* < 0.0001. **f** Kaplan–Meier plots of the overall survival rates of human ESCC patients (*n* = 81) with high and low expression of APC. *p*-Values were calculated using the two-tailed log-rank test. Source data are provided as a Source Data file.

To determine the clinical relevance of METTL3-suppressed APC expression, we performed TCGA data analyses and showed that *APC* mRNA expression was inversely correlated with *METTL3* mRNA expression in ESCC (Fig. 7a). In addition, we analysed 119 paired ESCC specimens and their adjacent normal tissues by gene expression profiles, and showed that APC expression was significantly downregulated in ESCC specimens compared to that in normal tissues (Fig. 7b). Consistent with the results obtained from TCGA data analyses, an inverse correlation between METTL3 protein expression and APC protein expression (Fig. 7c, d), and a positive correlation between METTL3 protein expression and β-catenin protein expression (Fig. 7c, e) were observed by IHC staining of 81 ESCC specimens. In addition, high expression of APC was significantly correlated with long overall survival time of patients with ESCC (Fig. 7f). These results suggest that downregulated APC expression correlates with upregulated METTL3 expression in human ESCC specimens and poor prognosis of ESCC patients.

## Discussion

**Fig. 8:**
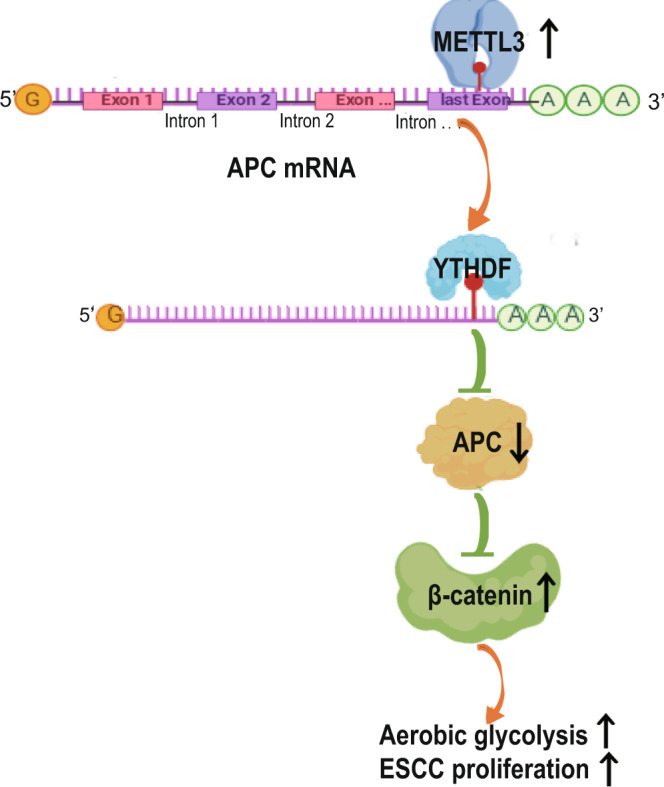
A mechanism underlying METTL3-promoted ESCC development. Overexpressed METTL3 increases the levels of the *APC* mRNA *N*^6^-methyladenosine, which recruits YTHDF to *APC* mRNA, to downregulate APC expression. Downregulated APC stabilizes β-catenin expression and promotes β-catenin-dependent gene expression for aerobic glycolysis, and ESCC cell proliferation and tumour growth.

*APC* is a critical tumour suppressor gene that plays an instrumental role in tumour development. APC mutations have been frequently detected in colorectal cancers but with a considerably lower frequency in some other types of cancers^6–10,53^. In addition to regulation by the gene mutation, whether *APC* is regulated at epitranscriptomic levels is unclear. Analysis of TCGA data and 200 paired ESCC specimens and their adjacent normal tissues showed that mRNA of *METTL3*, an important methyltransferase for m^6^A modification, is highly expressed in ESCC tissues compared to normal tissues. MeRIP and transcriptomal RNA sequencing revealed that METTL3 upregulates m^6^A modification in a large number of genes, including β-catenin destruction complex genes, such as *APC*. METTL3-enhanced *APC* mRNA m^6^A modification recruited YTHDF and elicited suppression of APC expression and subsequent enhanced expression of β-catenin, cyclin D1, c-Myc and c-Myc-regulated glycolytic genes, including *PKM2*. The reprogrammed expression of metabolic and cell cycle-promoting genes enhanced aerobic glycolysis, ESCC cell proliferation and tumour formation in mice (Fig. 8).

YTHDF mediate m^6^A-dependent RNA degradation^42,43^. We demonstrated that YTHDF bound METTL3-mediated m^6^A of *APC* mRNA and reduced *APC* expression, revealing a previously unknown mechanism by which the Wnt/β-catenin pathway is upregulated in cancer in an epitranscriptomal regulation-dependent manner. The clinical relevance and significance of our findings are evidenced by the correlation of upregulated METTL3 expression in human ESCC specimens with APC downregulation and poor prognosis in ESCC patients. The discovery of the critical regulation of APC by METTL3/YTHDF-coupled m^6^A regulation may provide promising approaches for the therapy of ESCC patients.

## Methods

### Materials

The following antibodies were purchased from Cell Signaling Technology (Danvers, MA): normal rabbit IgG (# 2729) (for immunoprecipitation, 1 : 5000 dilution), β-catenin (#8480) (for immunoblotting, 1 : 1000 dilution and IHC, 1 : 50 dilution), METTL3 (#86132) (for immunoblotting, 1 : 1000 dilution), METTL14 (#51104) (for immunoblotting, 1 : 1000 dilution), anti-rabbit secondary antibody (#8114) (for IHC, 1 : 50 dilution), cyclin D1 (#55506) (for immunoblotting, 1 : 1000 dilution), PKM2 (#4053) (for immunoblotting, 1 : 1000 dilution) and a mouse monoclonal antibody against α-Tubulin (#2144) (for immunoblotting, 1 : 5000 dilution). A rabbit antibody recognizing METTL3 (ab195352) (for IHC, 1 : 50 dilution and RIP), APC (ab15270) (for IHC, 1 : 100 dilution), APC (ab154906) (for immunoblotting, 1 : 1000 dilution) and c-Myc (ab32072, 1 : 1000 dilution) (for immunoblotting, 1 : 50 dilution) was purchased from Abcam (Cambridge, MA). Rabbit antibodies recognizing YTHDF1 (17479-1-AP) (for RIP, 1 : 50 dilution and immunoblotting, 1 : 1000 dilution) and YTHDF2 (24744-1-AP) (for RIP, 1 : 50 dilution and immunoblotting, 1 : 1000 dilution) and YTHDF3 (25537-1-AP) (for RIP, 1 : 50 dilution and immunoblotting, 1 : 1000 dilution) were purchased from Proteintech (IL, USA). The anti-m^6^A polyclonal antibody was purchased from Synaptic Systems. RIPA lysis and extraction buffer (89901) were purchased from Thermo Fisher Scientific (Waltham, MA). Nitro blue tetrazolium chloride (HY-15925) was purchased from MedChem Express (Monmouth Junction, NJ). Chromogen diaminobenzidine (DAB) staining was purchased from Cell Signaling Technology. Mayer’s haematoxylin and eosin (H&E) were purchased from Biogenex Laboratories (San Ramon, CA, USA). d-glucose ^13^C_6_ (389374) was purchased from Sigma-Aldrich (St. Louis, MO).

### Specimens and cell lines

Eighty-one pairs of frozen tissues were obtained from patients with ESCC, who underwent radical resections in the Department of Thoracic Surgery of the Cancer Hospital, Chinese Academy of Medical Sciences. The clinical features of the patients are summarized in Supplementary Table 1. All paired tumour and adjacent normal tissues used in this study were collected with informed consent. This study was approved by the Ethics Committee of the National Cancer Center/Cancer Hospital, Chinese Academy of Medical Sciences and Peking Union Medical College. TE1, TE10, KYSE30, KYSE70, KYSE140, KYSE150, KYSE180, KYSE410, KYSE450 and Het-1a cell lines were obtained from American Type Culture Collection.

### Tissue microarray construction

Formalin-fixed, paraffin-embedded tissues were obtained by surgical resection, archived after clinical use for pathological diagnosis and stained with Mayer’s H&E. Employing an automated tissue array instrument (Minicore^®^ 3, Alphelys, Plaisir, France), cancer tissue (diameter of 2 mm, selected by a pathologist) from each specimen was extracted and fixed into a paraffin block. After quality control, the TMA blocks were sectioned into 3 μm-thick slides for immunohistochemistry analysis.

### Immunohistochemistry

After deparaffinization, rehydration and antigen retrieval, TMA slides were incubated with primary rabbit anti-human METTL3 (dilution 1 : 500; Abcam Antibody; ab181064)^20^, primary rabbit anti-human APC (dilution 1 : 500; Abcam Antibody; ab154906)^21^ or nonspecific IgG (as a negative control) overnight at 4 °C. The slides were then incubated with anti-rabbit secondary antibody followed by chromogen DAB staining and haematoxylin counterstaining, and mounted with xylene-based medium. We quantitatively scored the tissue slides under a microscope according to the percentage of positive cells and staining intensity. A H-score (maximum score, 300) was assigned using the following formula: 3 × percentage of strongly staining + 2 × percentage of moderately staining + percentage of weakly staining^54^. Two pathologists (X.F. and S.S.) who were blinded to the clinical information independently validated the reproducibility of the scoring system.

### Cell culture

TE1, TE10, KYSE30, KYSE70, KYSE140, KYSE150, KYSE180, KYSE410, KYSE450 and Het-1a cells were grown in RPMI-1640 medium (Gibco) supplemented with 10% fetal bovine serum (Invitrogen) and 1% penicillin–streptomycin. Cells were cultured in 5% CO_2_ at 37 °C in a humidified incubator.

### RNA extraction and quantitative RT-PCR analysis

Total RNA was isolated with TRIzol reagent (Invitrogen, USA) according to the manufacturer’s instructions. RNA (1000 ng) was reverse-transcribed into cDNA with a RevertAid First Strand cDNA Synthesis kit (Thermo). SYBR Green-based quantitative reverse-transcriptase PCR was performed using a 7900HT fast real-time PCR system (Applied Biosystems/Life Technologies, Waltham, USA). The relative mRNA expression levels were calculated by the 2^−ΔΔCt^ method with normalization to ACTB and the PCR primers are listed in Supplementary Table 2.

### RNA m^6^A quantification

The m^6^A content of 200 ng of RNA extracted from tissues was measured by an EpiQuik m^6^A RNA methylation quantification kit (P-9005; Epigentek) according to the manufacturer’s instructions.

### Lentivirus production and infection

Plasmids containing transgenes and packaging plasmids were cotransfected into HEK 293T cells using Lipofectamine 3000 (Invitrogen, USA). Viruses were collected and concentrated after 48 h^55^. When tumour cells reached 50–60% confluence, we infected the cells with concentrated virus and then selected them by antibiotic treatment. The shRNA sequences are listed in Supplementary Table 3.

For the tetracycline-inducible (Tet-on) lentiviral expression, *METTL3* gene was cloned into the inducible Tet-on lentiviral vector (Beijing Syngentech Co., Ltd, Beijing, China). Tet-on lentivirus-infected KYSE450 cells were treated with or without a low (1 μg/ml) or a high dosage of tetracycline (2 μg/ml) (Sigma-Aldrich).

### Immunoblotting and immunoprecipitation analysis

Extraction of proteins with a modified buffer from cultured cells was followed by immunoprecipitation and immunoblotting with antibodies^56^.

### Cell proliferation assay

A total of 2 × 10^4^ cells were plated and counted 6 days (for KYSE180 cells) and 5 days (for KYSE450 cells) after seeding in RPMI-1640 medium with 10% bovine calf serum. KYSE180 and KYSE450 cell proliferation was measured using the Cell Counting Kit 8 (Dojindo) according to the manufacturer’s instructions. Data are presented as the means ± SD from three independent experiments.

### Colony formation assay

Cells were seeded in 60 mm plates (1000 cells/plate) and cultured for 10–12 days. The cells were then fixed with 4% formaldehyde, diluted in phosphate-buffered saline, stained with 2% crystal violet diluted in water and photographed.

### Dual-luciferase reporter assays

For m^6^A reporter assays, the DNA fragments of APC-Last Exon containing the WT m^6^A motifs, as well as the mutated motifs (m^6^A was replaced by T), were inserted into the Xhol site of pMIR-REPORT luciferase reporter vector. Dual-luciferase reporter assays were performed in HEK 293T cells^57^.

APC-Last Exon with WT m^6^A sites:

5′-TGAACTCTATTTCAGGAACCAAACAAAGTAAAGAAAACCAAGTATCCGCAAAAGGAACATGGAGAAAAATAAAAGAAAATGAATTTTCTCCCACAAATAGTACTTCTCAGACCGTTTCCTCAGGTGCTACAAATGGTGCTGAATCAAAGACT-3′

APC-Last Exon with the mutated m^6^A sites:

5′-TGATCTCTATTTCAGGAACCAAACAAAGTAAAGAAAACCAAGTATCCGCAAAAGGAACATGGAGAAAAATAAAAGAAAATGAATTTTCTCCCACAAATAGTACTTCTCAGTCCGTTTCCTCAGGTGCTACAAATGGTGCTGAATCAAAGTCT-3′

### Measurements of glucose consumption and lactate production

Cells were seeded in culture dishes and the medium was changed when cells reached 50% confluence. After incubation for 12–24 h, the culture medium was collected. The glucose levels were detected by a glucose colorimetric assay kit (#K606, BioVision), the lactate levels were detected by a lactate colorimetric assay kit (#K627, BioVision) according to the manufacturer’s instructions and values were calculated as previously described^51^.

### Animal experiments

For the subcutaneous implantation model, 1 × 10^6^ cells were injected subcutaneously into the flank regions of female BALB/c nude mice (4–5 weeks). The width (*W*) and length (*L*) of the tumours were measured every week, and the volume (*V*) of each tumour was calculated using the formula *V* = (*W*^2^ × *L*/2). All animal experiments were approved by the Animal Care and Use Committee of the Cancer Hospital of the Chinese Academy of Medical Sciences.

### RIP assays

A Magna RIP kit (17-700, Millipore, MA) was used to perform the RIP assays. Sufficient numbers of KYSE180 or KYSE450 cells were lysed by RIP lysis buffer and the supernatant of the RIP lysate was incubated with specific antibodies on beads overnight at 4 °C. After washing, RNA was extracted and analysed by reverse-transcriptase quantitative PCR.

### m^6^A-RIP and m^6^A sequencing (MeRIP-seq) assays

MeRIP-Seq was performed by Cloudseq Biotech, Inc. (Shanghai, China) according to the published procedure with slight modifications^15^. Briefly, fragmented mRNA was incubated with an anti-m^6^A polyclonal antibody (Synaptic Systems, 202003) in IPP buffer (10 mM Tris-HCl, 150 mM NaCl, 0.1% NP40, pH 7.4) for 2 h at 4 °C. The mixture was then immunoprecipitated by incubation with protein-A beads (Thermo Fisher) at 4 °C for an additional 2 h. The bound RNA was eluted from the beads with m^6^A (Berry & Associates, PR3732) in IPP buffer and then the RNA was extracted with TRIzol reagent (Thermo Fisher) following the manufacturer’s instructions. Purified RNA was used for RNA sequencing library generation with the NEBNext® Ultra™ RNA Library Prep kit (NEB). Both the input sample without immunoprecipitation and the m^6^A IP samples were subjected to 150 bp paired-end sequencing on an Illumina HiSeq 4000 sequencer.

### Sequencing data analysis

The paired-end reads were quality controlled by Q30 followed by removing of 3′-adapters and low-quality reads by cutadapt software (v1.9.3). Then, clean reads were aligned to the reference genome (UCSC HG19) by Hisat2 software (v2.0.4). The m^6^A peaks were predicted by Model-based Analysis of ChIP-Seq (MACS) software^58^. Motif enrichment analysis of predicted m^6^A peaks was performed by HOMER^59^ and distribution of m^6^A peaks was analysed by R package MetaPlotR^60^. Differentially methylated sites with a fold-change cut-off of ≥2 and false discovery rate cut-off of ≤0.00001 were identified by diffReps^61^. Raw counts of mRNA sequencing were got by HTSeq software (v0.9.1) and normalized by edgeR software. Then, the peaks were identified by MACS. GO analysis involving CC was performed with the database for annotation, visualization and integrated discovery. The *p*-value denotes the significance of GO term enrichment of the genes.

### MeRIP quantitative PCR

Total RNA was isolated with a miRNeasy kit (#217004, QIAGEN) with DNase I digestion. mRNA was extracted from total RNA using a GenElute mRNA Miniprep kit (MRN10, Sigma-Aldrich). Then, a Magna MeRIP m^6^A kit (#17-10499, Millipore) was used according to the manufacturer’s instructions. m^6^A enrichment was analysed by qPCR with specific primers and data were normalized to input. Primer sequences were as follows:

APC Forward: 5′-GAAGGAGTTAGAGGAGGGGC-3′;

APC Reverse: 5′-CCTCCTTGAGCCTCATCTGT-3′.

### Analysis of publicly available MeRIP-Seq and CLIP data

To study m^6^A level of *APC* mRNA in different types of cancer cells, the published MeRIP-Seq and CLIP data involved in this study were downloaded from GSE134380^30^, GSE87190^31^, GSE76367^17^, GSE128443^32^, GSE93911^33^, GSE102336^34^, GSE112795^35^ and GSE106122^36^. The percentage rank of *APC* m^6^A among all the gene transcripts with m^6^A in the indicated cancer cell types from published MeRIP-Seq and miCLIP data sets are shown in Supplementary Fig. 3c.

To analyse the m^6^A peak of *APC* mRNA in available miCLIP data (Fig. 3f), the published miCLIP data were downloaded from GSE71154^37^, GSE98623^38^, GSE122948^39^, GSE63753^40^ and GSE86336^41^. To study whether YTHDF1–3 bound *APC* mRNA, the published YTHDF1–3 CLIP data were downloaded from GSE63591^18^, GSE49339^44^, GSE86214^46^ and GSE78030^47^. Reads were analysed and aligned to the reference genome (UCSC HG19) by Hisat2 software (v2.0.4). Visual files were shown by IGV v2.8.3 in Fig. 3f and Supplementary Fig. 5a.

### ^13^C metabolic flux analysis and measurement of glycolytic intermediates

Steady-state labelling of glycolytic intermediates was accomplished by culturing KYSE450 cells (5 × 10^6^ cells/sample) in RPMI-1640 medium (Gibco) containing 2 g/L of d-glucose (U-^13^C_6_, 99%; Sigma-Aldrich) for 6 h. All treatments were conducted in quintuplicate. Intracellular metabolites were then extracted and measured. The Dionex Ultimate 3000 UPLC system was coupled to a TSQ Quantiva Ultra triple-quadrupole mass spectrometer (Thermo Fisher, CA), equipped with a heated electrospray ionization probe. The source parameters are as follows: capillary temperature: 350 °C; heater temperature: 300 °C; sheath gas flow rate: 35; auxiliary gas flow rate: 10. Tracefinder 3.2 (Thermo, USA) was applied for metabolite identification and peak integration.

### Statistical analysis

We used unpaired or paired Student’s *t*-tests to compare means between groups and all data are expressed as the mean ± SD. The survival analyses were performed using the Kaplan–Meier method to plot survival curves and the log-rank test to compare survival rates. The correlation between METTL3 and APC level was analysed by the Pearson correlation coefficient. *p*-values < 0.05 were considered to be significant. All statistical tests were two-sided.

### Reporting summary

Further information on research design is available in the Nature Research Reporting Summary linked to this article.

## Supplementary information

Supplementary Information

Description of Additional Supplementary Files

Supplementary Data 1

Reporting Summary

## Data Availability

The data of Figs. 1a and 7a, and Supplementary Fig. 1a are available in a public repository from the TCGA website. The clinical records and RNAseqV2 level 3 gene level ESCC data were downloaded from TCGA [http://xena.ucsc.edu/welcome-to-ucsc-xena]. Analysis data in Fig. 1a is available in UALCAN [http://ualcan.path.uab.edu/cgi-bin/TCGAExResultNew2.pl?genenam=METTL3&ctype=ESCA]^62^. The clinical records and genome-wide gene expression profiles (Affymetrix GeneChip Human Exon 1.0 ST arrays) of a total of 119 paired ESCC data sets were downloaded from the Gene Expression Omnibus with accession number GSE53625^63^. Gene transcription estimates for each gene were analysed using Robust Multiarray Average (RMA) software. The MeRIP-seq and mRNA-seq data have been deposited into the Gene Expression Omnibus repository under accession number GSE154555. The remaining data are available within the article, Supplementary Information or available from the authors upon request. Source data are provided with this paper.

## Source data

Source Data

## References

[1] van de Wetering M (2002). The beta-catenin/TCF-4 complex imposes a crypt progenitor phenotype on colorectal cancer cells. Cell.

[2] Nguyen H, Rendl M, Fuchs E (2006). Tcf3 governs stem cell features and represses cell fate determination in skin. Cell.

[3] Kirstetter P, Anderson K, Porse BT, Jacobsen SE, Nerlov C (2006). Activation of the canonical Wnt pathway leads to loss of hematopoietic stem cell repopulation and multilineage differentiation block. Nat. Immunol..

[4] Korinek V (1997). Constitutive transcriptional activation by a beta-catenin-Tcf complex in APC-/- colon carcinoma. Science.

[5] Fang D (2007). Phosphorylation of beta-catenin by AKT promotes beta-catenin transcriptional activity. J. Biol. Chem..

[6] Cancer Genome Atlas Network. Comprehensive molecular characterization of human colon and rectal cancer. *Nature* **487**, 330–337 (2012). 10.1038/nature11252 PMC3401966 22810696

[7] Vasaikar S (2019). Proteogenomic analysis of human colon cancer reveals new therapeutic opportunities. Cell.

[8] Yaeger R (2018). Clinical sequencing defines the genomic landscape of metastatic colorectal cancer. Cancer Cell.

[9] Lin DC (2014). Genomic and molecular characterization of esophageal squamous cell carcinoma. Nat. Genet..

[10] Song Y (2014). Identification of genomic alterations in oesophageal squamous cell cancer. Nature.

[11] Gilbert WV, Bell TA, Schaening C (2016). Messenger RNA modifications: form, distribution, and function. Science.

[12] Zhao BS, Roundtree IA, He C (2017). Post-transcriptional gene regulation by mRNA modifications. Nat. Rev. Mol. Cell Biol..

[13] Deng X (2015). Widespread occurrence of N6-methyladenosine in bacterial mRNA. Nucleic Acids Res..

[14] Marbaniang CN, Vogel J (2016). Emerging roles of RNA modifications in bacteria. Curr. Opin. Microbiol..

[15] Meyer KD (2012). Comprehensive analysis of mRNA methylation reveals enrichment in 3’ UTRs and near stop codons. Cell.

[16] Fustin JM (2013). RNA-methylation-dependent RNA processing controls the speed of the circadian clock. Cell.

[17] Lin S, Choe J, Du P, Triboulet R, Gregory RI (2016). The m(6)A methyltransferase METTL3 promotes translation in human cancer cells. Mol. cell.

[18] Wang X (2015). N(6)-methyladenosine modulates messenger RNA translation efficiency. Cell.

[19] Liu J (2020). N (6)-methyladenosine of chromosome-associated regulatory RNA regulates chromatin state and transcription. Science.

[20] Chen T (2015). m(6)A RNA methylation is regulated by microRNAs and promotes reprogramming to pluripotency. Cell Stem Cell.

[21] Chen XY, Zhang J, Zhu JS (2019). The role of m(6)A RNA methylation in human cancer. Mol. Cancer.

[22] Yang Y, Hsu PJ, Chen YS, Yang YG (2018). Dynamic transcriptomic m(6)A decoration: writers, erasers, readers and functions in RNA metabolism. Cell Res..

[23] Li T (2019). METTL3 facilitates tumor progression via an m(6)A-IGF2BP2-dependent mechanism in colorectal carcinoma. Mol. Cancer.

[24] Chen M (2018). RNA N6-methyladenosine methyltransferase-like 3 promotes liver cancer progression through YTHDF2-dependent posttranscriptional silencing of SOCS2. Hepatology.

[25] Li Z (2017). FTO plays an oncogenic role in acute myeloid leukemia as a N(6)-methyladenosine RNA demethylase. Cancer Cell.

[26] Jemal A (2011). Global cancer statistics. CA Cancer J. Clin..

[27] Siegel RL, Miller KD, Jemal A (2016). Cancer statistics, 2016. CA Cancer J. Clin..

[28] Zeng H (2016). Esophageal cancer statistics in China, 2011: estimates based on 177 cancer registries. Thorac. Cancer.

[29] Grozhik AV, Jaffrey SR (2018). Distinguishing RNA modifications from noise in epitranscriptome maps. Nat. Chem. Biol..

[30] Zaccara S, Jaffrey SR (2020). A unified model for the function of YTHDF proteins in regulating m(6)A-modified mRNA. Cell.

[31] Su R (2018). R-2HG exhibits anti-tumor activity by targeting FTO/m(6)A/MYC/CEBPA signaling. Cell.

[32] Song H (2020). SFPQ is an FTO-binding protein that facilitates the demethylation substrate preference. Cell Chem. Biol..

[33] Liu J (2018). m(6)A mRNA methylation regulates AKT activity to promote the proliferation and tumorigenicity of endometrial cancer. Nat. Cell Biol..

[34] Ma H (2019). N(6-)Methyladenosine methyltransferase ZCCHC4 mediates ribosomal RNA methylation. Nat. Chem. Biol..

[35] Li Z (2020). N(6)-methyladenosine regulates glycolysis of cancer cells through PDK4. Nat. Commun..

[36] Kuppers DA (2019). N(6)-methyladenosine mRNA marking promotes selective translation of regulons required for human erythropoiesis. Nat. Commun..

[37] Ke S (2015). A majority of m6A residues are in the last exons, allowing the potential for 3’ UTR regulation. Genes Dev..

[38] Vu LP (2017). The N(6)-methyladenosine (m(6)A)-forming enzyme METTL3 controls myeloid differentiation of normal hematopoietic and leukemia cells. Nat. Med..

[39] Boulias K (2019). Identification of the m(6)Am methyltransferase PCIF1 reveals the location and functions of m(6)Am in the transcriptome. Mol. Cell.

[40] Linder B (2015). Single-nucleotide-resolution mapping of m6A and m6Am throughout the transcriptome. Nat. Methods.

[41] Ke S (2017). m(6)A mRNA modifications are deposited in nascent pre-mRNA and are not required for splicing but do specify cytoplasmic turnover. Genes Dev..

[42] Du H (2016). YTHDF2 destabilizes m(6)A-containing RNA through direct recruitment of the CCR4-NOT deadenylase complex. Nat. Commun..

[43] Wang X, He C (2014). Reading RNA methylation codes through methyl-specific binding proteins. RNA Biol..

[44] Wang X (2014). N6-methyladenosine-dependent regulation of messenger RNA stability. Nature.

[45] Jin D (2020). m(6)A demethylase ALKBH5 inhibits tumor growth and metastasis by reducing YTHDFs-mediated YAP expression and inhibiting miR-107/LATS2-mediated YAP activity in NSCLC. Mol. Cancer.

[46] Shi H (2017). YTHDF3 facilitates translation and decay of N(6)-methyladenosine-modified RNA. Cell Res..

[47] Patil DP (2016). m(6)A RNA methylation promotes XIST-mediated transcriptional repression. Nature.

[48] Yang W (2012). PKM2 phosphorylates histone H3 and promotes gene transcription and tumorigenesis. Cell.

[49] Yang W (2011). Nuclear PKM2 regulates β-catenin transactivation upon EGFR activation. Nature.

[50] Yang W, Lu Z (2013). Nuclear PKM2 regulates the Warburg effect. Cell Cycle.

[51] Yang W, Lu Z (2013). Regulation and function of pyruvate kinase M2 in cancer. Cancer Lett..

[52] Yang W (2012). ERK1/2-dependent phosphorylation and nuclear translocation of PKM2 promotes the Warburg effect. Nat. cell Biol..

[53] Peng H, Zhong XY, Liu KP, Li SM (2009). Expression and significance of adenomatous polyposis coli, beta-catenin, E-cadherin and cyclin D1 in esophageal squamous cell carcinoma assessed by tissue microarray. Ai Zheng.

[54] Hirsch FR (2003). Epidermal growth factor receptor in non-small-cell lung carcinomas: correlation between gene copy number and protein expression and impact on prognosis. J. Clin. Oncol..

[55] Kutner RH, Zhang XY, Reiser J (2009). Production, concentration and titration of pseudotyped HIV-1-based lentiviral vectors. Nat. Protoc..

[56] Lu Z (1998). Activation of protein kinase C triggers its ubiquitination and degradation. Mol. Cell. Biol..

[57] Li Y (2018). Novel long noncoding RNA NMR promotes tumor progression via NSUN2 and BPTF in esophageal squamous cell carcinoma. Cancer Lett..

[58] Zhang Y (2008). Model-based analysis of ChIP-Seq (MACS). Genome Biol..

[59] Heinz S (2010). Simple combinations of lineage-determining transcription factors prime cis-regulatory elements required for macrophage and B cell identities. Mol. Cell.

[60] Olarerin-George AO, Jaffrey SR (2017). MetaPlotR: a Perl/R pipeline for plotting metagenes of nucleotide modifications and other transcriptomic sites. Bioinformatics.

[61] Shen L (2013). diffReps: detecting differential chromatin modification sites from ChIP-seq data with biological replicates. PLoS ONE.

[62] Chandrashekar, D. S. et al. UALCAN: A Portal for Facilitating Tumor Subgroup Gene Expression and Survival Analyses. *Neoplasia* **19**, 649–658 (2017). 10.1016/j.neo.2017.05.002 PMC5516091 28732212

[63] Li J (2014). LncRNA profile study reveals a three-lncRNA signature associated with the survival of patients with oesophageal squamous cell carcinoma. Gut.

