# Author Correction: METTL3 promotes tumour development by decreasing APC expression mediated by *APC* mRNA *N*^6^-methyladenosine-dependent YTHDF binding

**DOI:** 10.1038/s41467-021-24860-9

**Published:** 2021-07-20

**Authors:** Wei Wang, Fei Shao, Xueying Yang, Juhong Wang, Rongxuan Zhu, Yannan Yang, Gaoxiang Zhao, Dong Guo, Yingli Sun, Jie Wang, Qi Xue, Shugeng Gao, Yibo Gao, Jie He, Zhimin Lu

**Affiliations:** 1 Department of Thoracic Surgery, National Cancer Center/National Clinical Research Center for Cancer/Cancer Hospital, Chinese Academy of Medical Sciences and Peking Union Medical College, Beijing, China; 2 Department of Medical Oncology, National Cancer Center/National Clinical Research Center for Cancer/Cancer Hospital, Chinese Academy of Medical Sciences and Peking Union Medical College, Beijing, China; 3 Affiliated Hospital of Qingdao University and Qingdao Cancer Institute, Qingdao, Shandong China; 4 Zhejiang Provincial Key Laboratory of Pancreatic Disease, The First Affiliated Hospital and Institute of Translational Medicine, Zhejiang University School of Medicine, Hangzhou, China; 5 National Cancer Center/National Clinical Research Center for Cancer/Cancer Hospital and Shenzhen Hospital, Chinese Academy of Medical Sciences and Peking Union Medical College, Shenzhen, China; 6 State Key Laboratory of Molecular Oncology, National Cancer Center/National Clinical Research Center for Cancer/Cancer Hospital, Chinese Academy of Medical Sciences and Peking Union Medical College, Beijing, China; 7 Zhejiang University Cancer Center, Hangzhou, China

**Keywords:** Cancer, Cell biology, Molecular biology, Gastroenterology, Medical research

Correction to: *Nature Communications* 10.1038/s41467-021-23501-5, published online 21 June 2021

The original version of this Article contained an error in the author’s affiliations.

The affiliation of Zhimin Lu with the Department of Thoracic Surgery, National Cancer Center/National Clinical Research Center for Cancer/Cancer Hospital, Chinese Academy of Medical Sciences and Peking Union Medical College, Beijing, China, was inadvertently omitted.

This has now been corrected in both the PDF and HTML versions of the Article.

