# Supplementary material for: METTL3 promotes tumour development by decreasing APC expression mediated by APC mRNA N6-methyladenosine-dependent YTHDF binding
Source: Nat Commun. 2021 Jun 21;12:3803. doi: 10.1038/s41467-021-23501-5 (PMC8217513; doi:10.1038/s41467-021-23501-5)
Supplement: Supplementary file 1 — Supplementary Information [file 41467_2021_23501_MOESM1_ESM.pdf]

Supplementary Figure 1

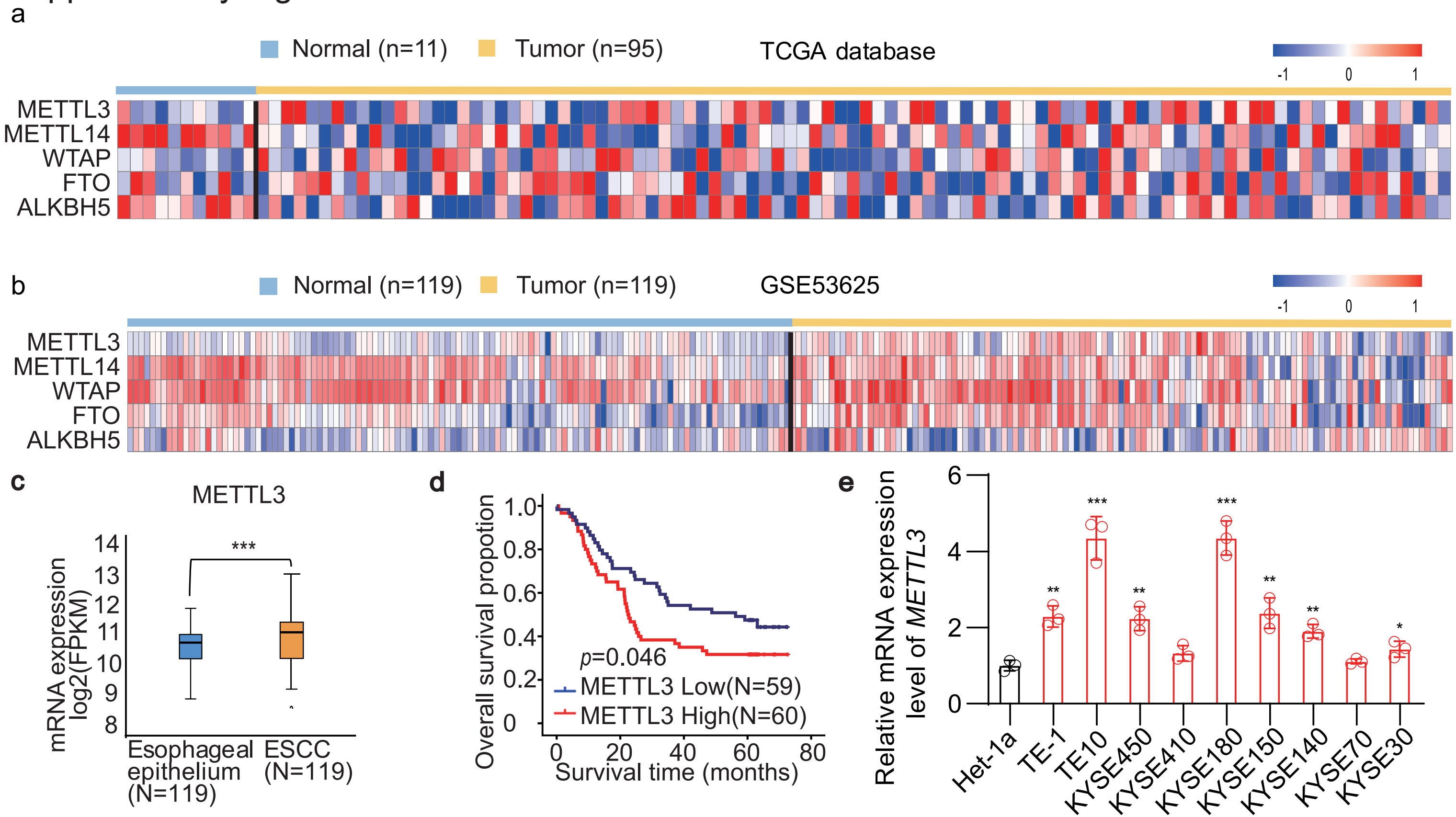

Supplementary Fig. 1 Upregulated METTL3 is correlated with poor survival in ESCC patients

(a) The mRNA expression levels of m^6A regulators in ESCC tissues (*n* = 95) and their adjacent normal tissues (*n* = 11) (from the TCGA dataset).

(b) The mRNA expression levels of m^6A regulators in 119 ESCC tissues and their adjacent normal epithelial tissues (from the GSE53625 dataset).

(c) The relative mRNA expression levels of *METTL3* in 119 ESCC tissues and their adjacent normal epithelial tissues (from the GSE53625 dataset) were analysed. ***p = 2.60E-06 based on two-tailed Student's t test. For boxplot, box boundaries correspond to 1st and 3rd quartiles; whiskers extend to a maximum of 1.5x the inter-quartile range.

(d) Kaplan-Meier method was used to plot survival cures in human ESCC specimens (*n* = 119) with high (*n* = 60) and low (*n* = 59) expression levels of METTL3. The two-tailed log-rank test was used to compare survival rate.

(e) Relative mRNA expression levels of METTL3 in 9 ESCC cell lines and Het-1a immortalized normal human esophageal epithelial cells were determined by qRT-PCR. Data represent the means ± SD of triplicate samples. * p= 0.0397, ** p = 0.0019, 0.0034, 0.0049, 0.0024 (left to right), *** p =0.0006, 0.0002 (left to right) based on two-tailed Student's t test.

Source data are provided as a Source Data file.

Supplementary Figure 2

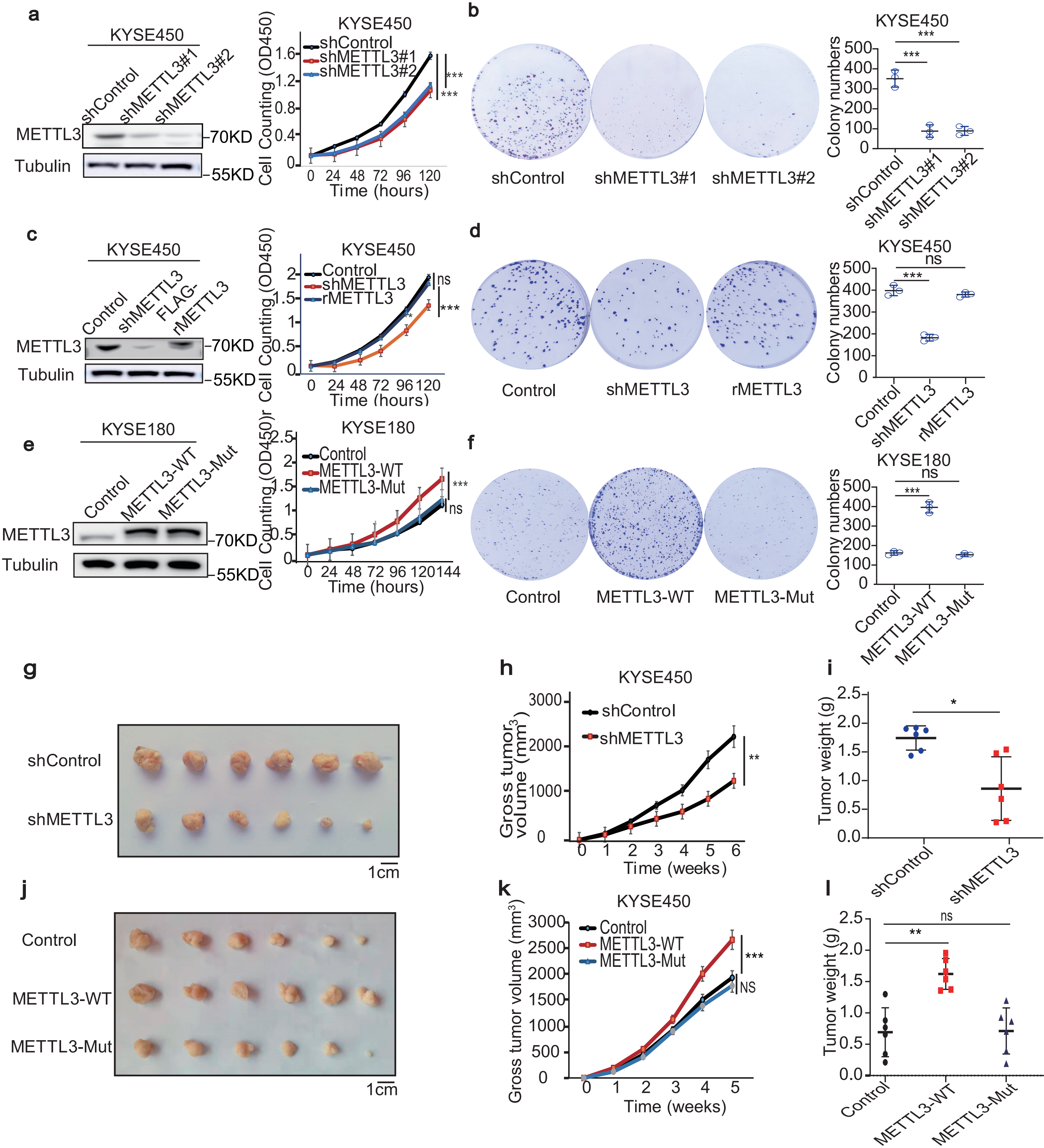

Supplementary Fig. 2. METTL3 promotes ESCC cell proliferation and tumour growth in mice

(**a**) KYSE450 cells were stably transfected with a control vector or two different METTL3 shRNAs. Immunoblotting analyses with the indicated antibodies were performed. KYSE450 cells with or without METTL3 depletion were cultured for the indicated periods of time and were harvested for cell counting. Data represent the means ± SD of triplicate samples. ***p =0.0003, 1.47E-05 (left to right) based on two-tailed Student's t test.

(**b**) KYSE450 cells with or without METTL3 depletion were cultured for 10 days. The stained colony numbers were counted. Data represent the means ± SD of triplicate samples. ***p =0.0009, 0.0007 (left to right).

(**c**) KYSE450 cells with or without METTL3 depletion and with or without reconstituted expression of RNAi-resistant Flag-rMETTL3 were analysed by immunoblotting analyses with the indicated antibodies. KYSE450 cells with or without METTL3 depletion and with or without reconstituted expression of RNAi-resistant Flag-rMETTL3 were cultured for the indicated periods of time and were harvested for cell counting. Data represent the means ± SD of triplicate samples. ***p =0.0002 based on two-tailed Student's t test. ns, not significant.

(**d**) KYSE450 cells with or without METTL3 depletion and with or without reconstituted expression of RNAi-resistant Flag-rMETTL3 were cultured for 10 days. The stained colony numbers were counted. Data represent the means ± SD of triplicate samples. ***p =0.0002 based

on two-tailed Student’s t test. ns, not significant.

(**e**) KYSE180 cells with or without expressing wild-type METTL3 or an inactive METTL3 mutant were analysed by immunoblotting analyses with the indicated antibodies or cultured for the indicated periods of time for cell counting. Data represent the means ± SD of triplicate samples. ***$p = 0.0001$ based on two-tailed Student’s t test. ns, not significant.

(**f**) METTL3 cells with or without expressing WT METTL3 or an inactive METTL3 mutant were cultured for 12 days. The stained colony numbers were counted. Data represent the means ± SD of triplicate samples. ***$p = 0.0002$ based on two-tailed Student’s t test. ns, not significant.

(g-i) KYSE450 cells with or without METTL3 depletion were subcutaneously injected into the flank regions of nude mice ($n = 6$). Six weeks later, tumour sizes **(g),** volumes **(h),** and weight (**i**) were calculated. scale bar, 1 cm. Data represent the means ± SD of 6 mice in each group. **p= 0.0013 (**h**), *$p = 0.0104$ **(i)** based on two-tailed Student’s t test.

(j-l) KYSE450 cells cells with or without expressing wild-type METTL3 or an inactive METTL3 mutant were subcutaneously injected into the flank regions of nude mice ($n = 6$). Five weeks later, tumour sizes **(j),** volumes **(k),** and weight (**l**) were measured. scale bar, 1 cm. Data represent the means ± SD of 6 mice in each group. ***p= 2.91E-05 (**k**), **$p = 0.0013$ (**l**) based on two-tailed Student’s t test. ns, not significant.

Source data are provided as a Source Data file.

Supplementary Figure 3

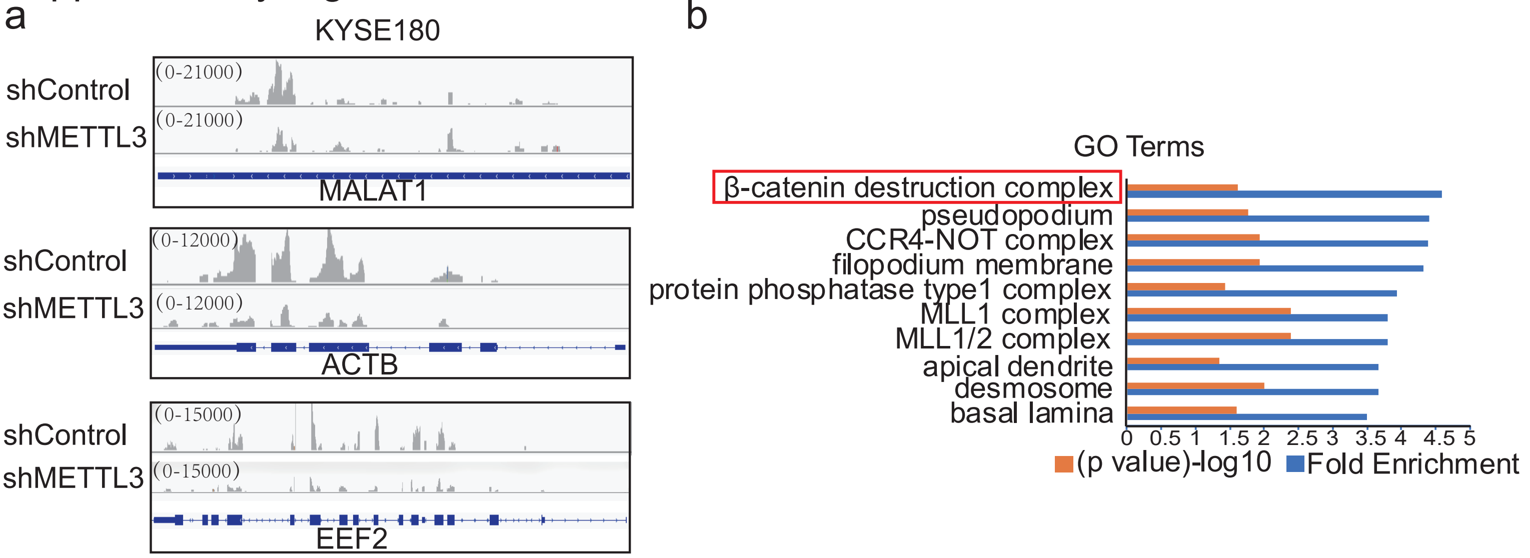

c

GSE number	Cell line	Cancer type	Ranking of APC methylation (%)
GSE134380	HeLa	Cervical carcinoma	0.014780405
GSE87190	NOMO-1 AML	Leukemia	4.787328077
GSE76367	H1299	Lung cancer	5.226615236
GSE128443	HeLa	Cervical carcinoma	5.782800149
GSE93911	HEC-1-A	Endometrial adenocarcinoma	6.163233127
GSE87190	MA9.3ITD AML	Leukemia	6.636642272
GSE102336	HepG2	Liver cancer	7.580113963
GSE112795	HeLa	Cervical carcinoma	7.705790704
GSE106122	HEL	Erythroleukemia	9.464831804

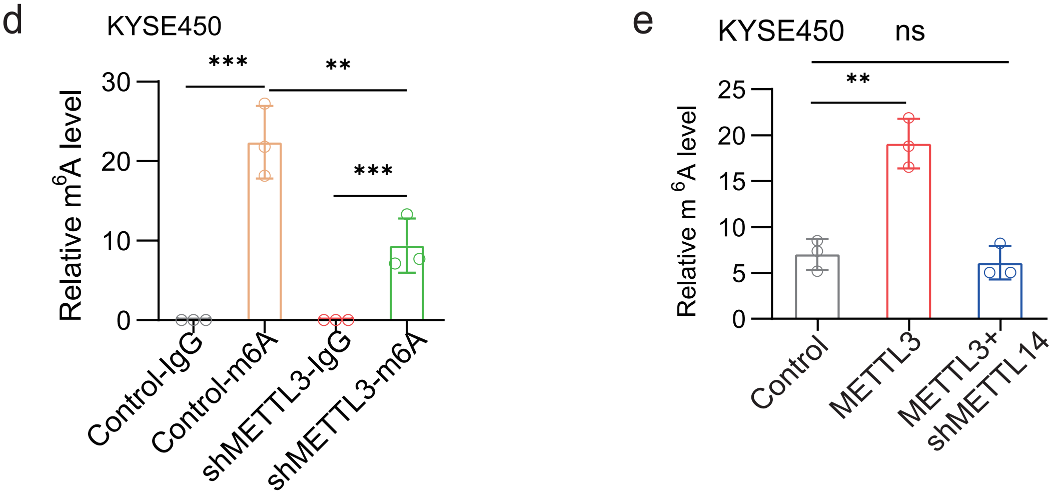

Supplementary Fig. 3 METTL3 mediated m^{6}A upregulation on *APC* mRNA.

(a) The m^{6}A peaks of the indicated transcripts from MeRIP-seq of KYSE180 cells with or without METTL3 depletion are displayed.

(b) MeRIP-seq of KYSE180 cells with or without METTL3 depletion was conducted. The GO analyses of CC terms based on MeRIP-seq showed the most significant GO terms and the *p* values in KYSE180 cells with or without METTL3 depletion.

(c) The percentage rank of APC m^{6}A among all the gene transcripts with m^{6}A in the indicated cancer cell types from published MeRIP-Seq and miCLIP data sets are shown.

(d) Methylated RNA in KYSE450 cells with or without METTL3 depletion was immunoprecipitated with an m^{6}A antibody followed by qPCR analyses with primers against *APC* mRNA. Data represent the means ± SD of triplicate samples. $^{**}p$ =0.0011, $^{***}p$ =8.56E-05, 9.92E-05 (left to right) based on two-tailed Student's t test.

(e) Methylated RNA in KYSE450 cells transfected with or without a vector expressing METTL3 or vectors expressing METTL3 and METTL14 shRNA was immunoprecipitated with an m^{6}A antibody followed by qPCR analyses with primers against *APC* mRNA. Data represent the means ± SD of triplicate samples. $^{**}p$ = 0.0028 based on two-tailed Student's t test. ns, not significant.

Source data are provided as a Source Data file.

Supplementary Figure 4

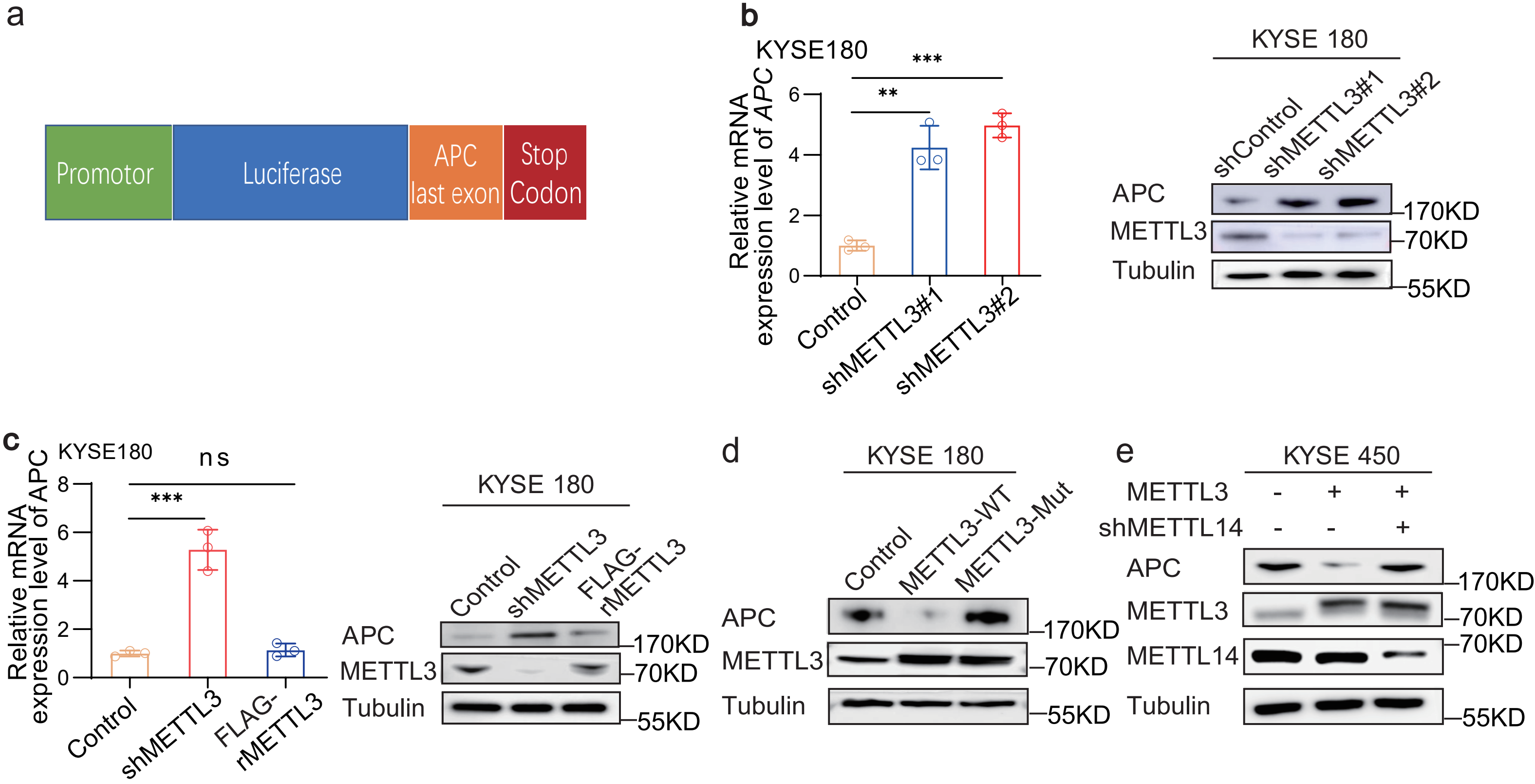

Supplementary Fig. 4. METTL3-dependent m^{6}A upregulation on *APC* mRNA suppresses APC expression

(**a**) *APC* coding sequences (CDSs) containing WT m^{6}A motifs or mutated m^{6}A motifs (A-T) were inserted into the Xhol site immediately before the stop codon of the luciferase gene.

(**b**) The relative mRNA expression levels of *APC* in KYSE180 cells with or without METTL3 depletion were determined by qPCR. Data represent the means ± SD of triplicate samples. $**p = 0.0016$, $***p = 8.96E-05$ based on two-tailed Student's t test. The protein expression levels of *APC* in KYSE450 cells with or without METTL3 depletion were analysed by immunoblotting analyses with the indicated antibodies.

(**c**) The relative mRNA and protein expression levels of *APC* in KYSE180 cells with or without METTL3 depletion or with reconstituted expression of RNAi-resistant Flag-rMETTL3 were determined by qPCR and immunoblotting analyses with the indicated antibodies, respectively. Data represent the means ± SD of triplicate samples. $***p = 0.0009$ based on two-tailed Student's t test. ns, not significant.

(**d**) KYSE180 cells with or without expressing WT METTL3 or an inactive METTL3 mutant were analysed by immunoblotting analyses with the indicated antibodies three times with similar results.

(**e**) KYSE450 cells were transfected with or without a vector expressing METTL3 or vectors expressing METTL3 and METTL14 shRNA. Immunoblotting analyses were performed with the indicated antibodies three times with similar results. Source data are provided

Source data are provided as a Source Data file.

Supplementary Figure 5

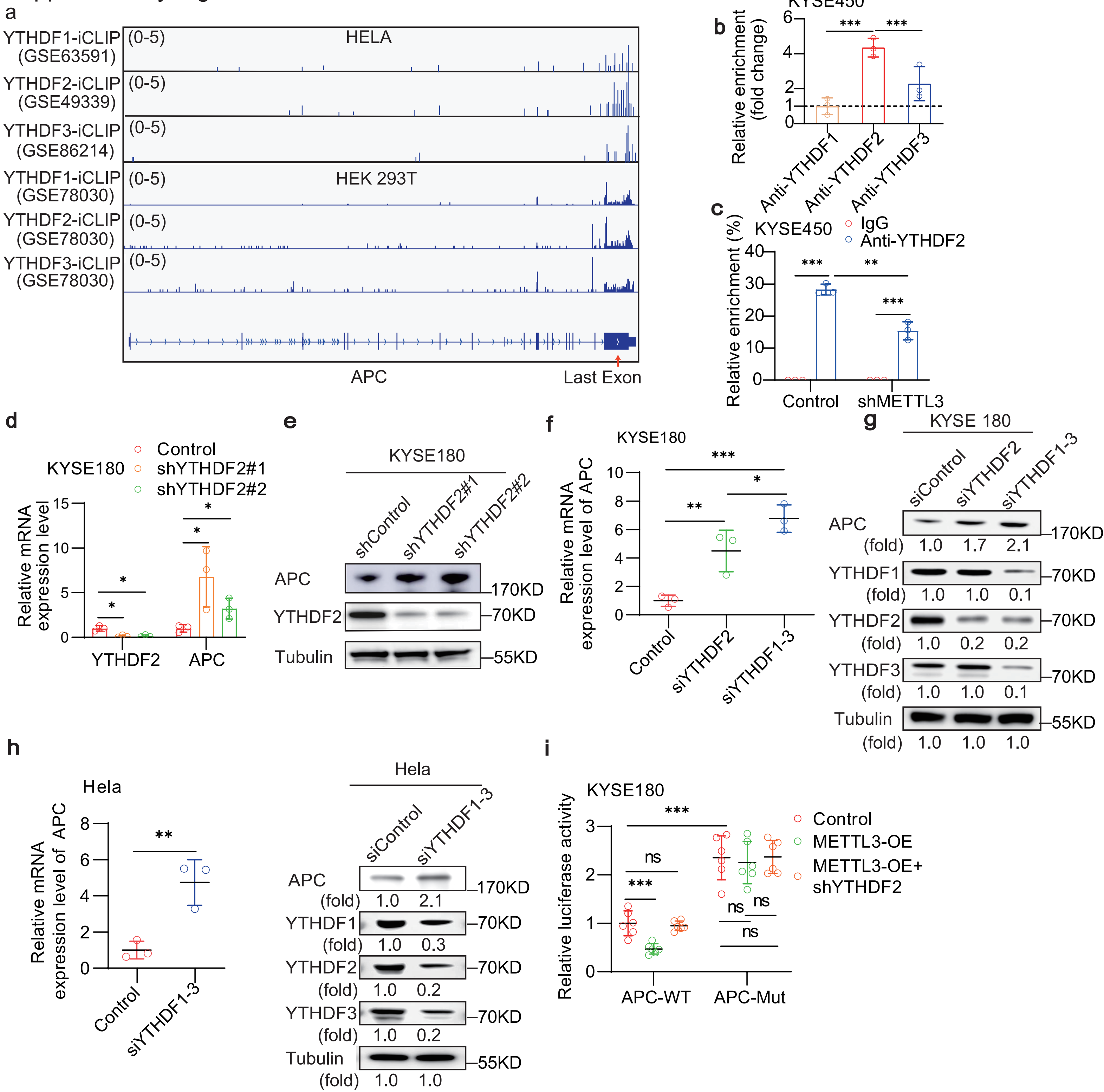

Supplementary Fig. 5 METTL3-enhanced m6A of *APC* mRNA and subsequent binding of YTHDF2 suppresses APC expression.

(a) Published iCLIP results showed the binding of YTHDF1, YTHDF2, and YTHDF3 on *APC*. All these YTHDF1-3 CLIP peaks were distribution on last exon of *APC*.

(b) RIP analyses of KYSE450 cells were performed with antibodies against YTHDF1, YTHDF2 or YTHDF3, followed by qPCR analyses with primers against *APC* mRNA. Data represent the means ± SD of triplicate samples. **p* = 0.0323, ***p* = 0.0012 based on two-tailed Student's t test.

(c) RIP analyses of KYSE450 cells with or without METTL3 depletion were performed with an anti-YTHDF2 antibody followed by qPCR analyses with primers against *APC* mRNA. Data represent the means ± SD of triplicate samples. ***p* = 0.0024, ****p* = 8.54E-06 and 0.0007 (left to right) based on two-tailed Student's t test.

(d) The relative mRNA expression levels of *APC* in KYSE180 cells with or without YTHDF2 depletion were determined by qPCR. Data represent the means ± SD of triplicate samples. **p* = 0.0102, 0.0113, 0.0421, 0.0355 (left to right) based on two-tailed Student's t test.

(e) The protein expression levels of *APC* in KYSE180 cells with or without YTHDF2 depletion were analysed by immunoblotting analyses with the indicated antibodies.

(f) KYSE450 cells were transfected with or without a YTHDF2 siRNA or combination of YTHDF1-3 siRNAs. The relative mRNA expression levels of *APC* were measured using

quantitative qPCR. Data represent the means ± SD of triplicate samples. *$p = 0.037$, **$p = 0.0064$, ***$p = 0.0007$ based on two-tailed Student's t test.

(g) KYSE450 cells were transfected with or without a YTHDF2 siRNA or combination of YTHDF1-3 siRNAs. Immunoblotting analyses were performed with the indicated antibodies.

(h) HeLa cells were transfected with or without combination of YTHDF1-3 siRNAs. The relative mRNA expression levels of *APC* were measured using quantitative qPCR. Data represent the means ± SD of triplicate samples. **$p = 0.0088$ based on two-tailed Student's t test. Immunoblotting analyses were performed with the indicated antibodies.

(i) Luciferase vectors with WT or mutated m^6A nucleotides in the *APC* gene were transfected into KYSE180 cells with or without METTL3 overexpression or combined METTL3 overexpression and YTHDF2 depletion. Luciferase activity was measured. Data represent the means ± SD of triplicate samples. ***$p = 0.0009$, 8.47E-05 (left to right) based on two-tailed Student's t test. ns, not significant.

Source data are provided as a Source Data file.

Supplementary Figure 6

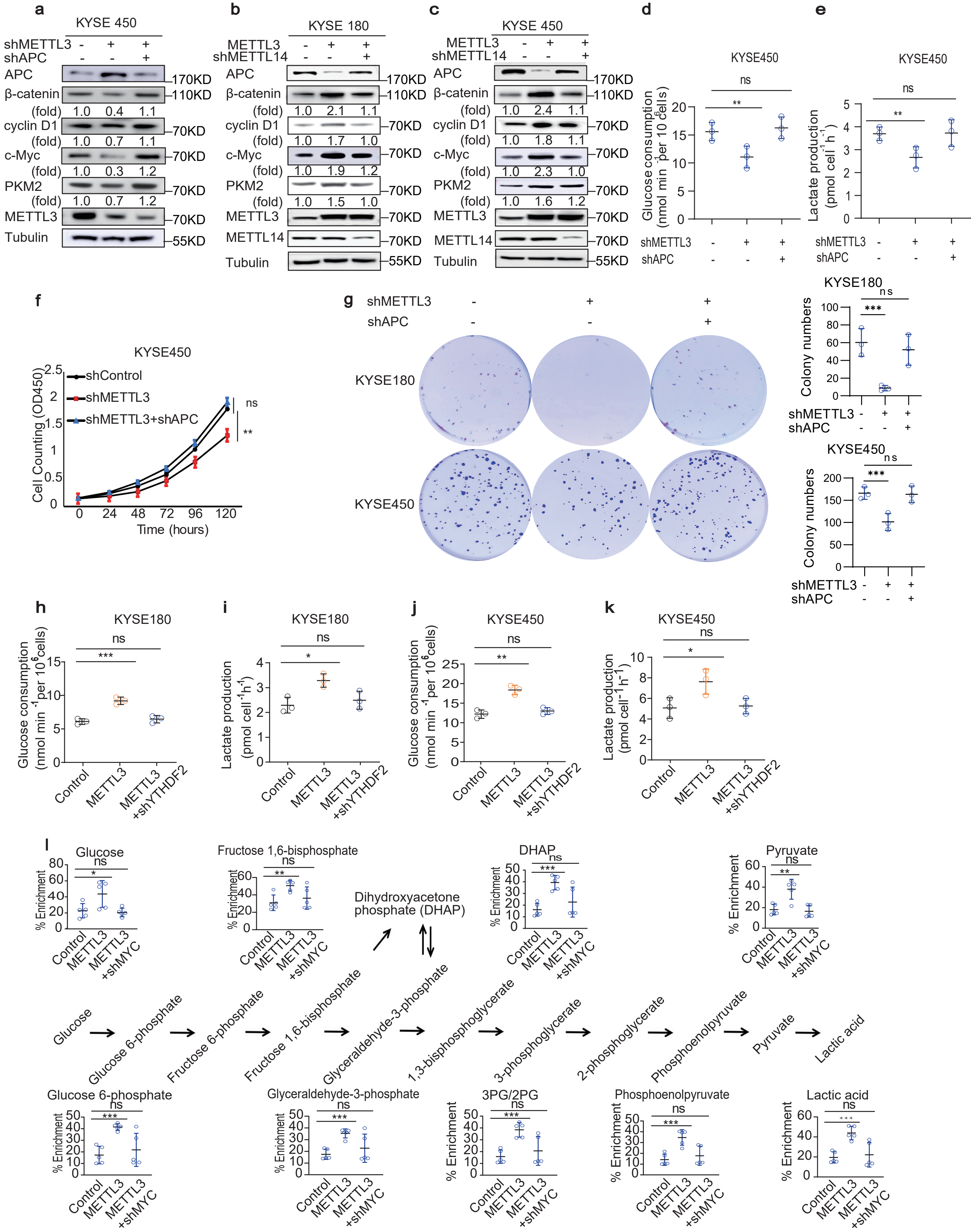

Supplementary Fig. 6 METTL3 reduces APC expression and promotes β-catenin-mediated downstream gene expression, aerobic glycolysis, and ESCC cell proliferation

(a) Immunoblotting analyses of KYSE450 cells with or without *METTL3* shRNA expression or combined *METTL3* shRNA and *APC* shRNA expression were performed with the indicated antibodies three times with similar results.

(b) KYSE180 cells were transfected with or without a vector expressing METTL3 or vectors expressing METTL3 and METTL14 shRNA. Immunoblotting analyses were performed with the indicated antibodies three times with similar results.

(c) KYSE450 cells were transfected with or without a vector expressing METTL3 or vectors expressing METTL3 and METTL14 shRNA. Immunoblotting analyses were performed with the indicated antibodies three times with similar results.

(**d**, **e**) Glucose consumption **(d)** and lactate production **(e)** of KYSE450 cells with or without *METTL3* shRNA expression or combined *METTL3* shRNA and *APC* shRNA expression were determined. Data represent the means ± SD of triplicate samples. $^{**}p$ = 0.0030 **(d),** 0.0091 **(e)** based on two-tailed Student's t test. ns, not significant.

(**f**) KYSE450 cells with or without *METTL3* shRNA expression or combined *METTL3* shRNA and *APC* shRNA expression were cultured for the indicated periods of time and were harvested for cell counting. Data represent the means ± SD of triplicate samples. $^{**}p$ = 0.0027 based on two-tailed Student's t test. ns, not significant.

(**g**) KYSE180 and KYSE450 cells with or without METTL3 depletion were cultured for 12 days (KYSE180) or 10 days (KYSE450). The stained colony numbers were counted. Data represent the means ± SD of triplicate samples. ***p = 0.0004, = 0.0007 (up to down) based on two-tailed Student's t test. ns, not significant.

(**h and i**) Glucose consumption **(h)** and lactate production **(i)** of KYSE180 cells with or without *METTL3* shRNA expression or combined *METTL3* shRNA and *YTHDF2* shRNA expression were determined. Data represent the means ± SD of triplicate samples. *p = 0.0144 **(i)**, ***p = 9.20E-05 (**h**) based on two-tailed Student's t test. ns, not significant.

(**j and k**) Glucose consumption **(j)** and lactate production **(k)** of KYSE450 cells with or without *METTL3* shRNA expression or combined *METTL3* shRNA and *YTHDF2* shRNA expression were determined. Data represent the means ± SD of triplicate samples. *p = 0.0488 (**k**), **p = 0.0027 (**j**) based on two-tailed Student's t test. ns, not significant.

(**l**) KYSE450 cells with or without METTL3 overexpression or combined METTL3 overexpression and MYC depletion, were incubated with $^{13}C_6$-glucose for 6 hours. ^{13}C enrichment of glycolytic intermediates were measured by gas chromatography (GC)-mass spectrometer. Data represent the means ± SD of quintuplicate samples. *p = 0.0389; **p = 0.0037, 0.0037 (left to right); ***p = 0.0002, 0.0002, 0.0001, 0.0003, 0.0009, 0.0002 (up to down, left to right) based on two-tailed Student's t test. ns, not significant.

Source data are provided as a Source Data file.

Supplementary Figure 7

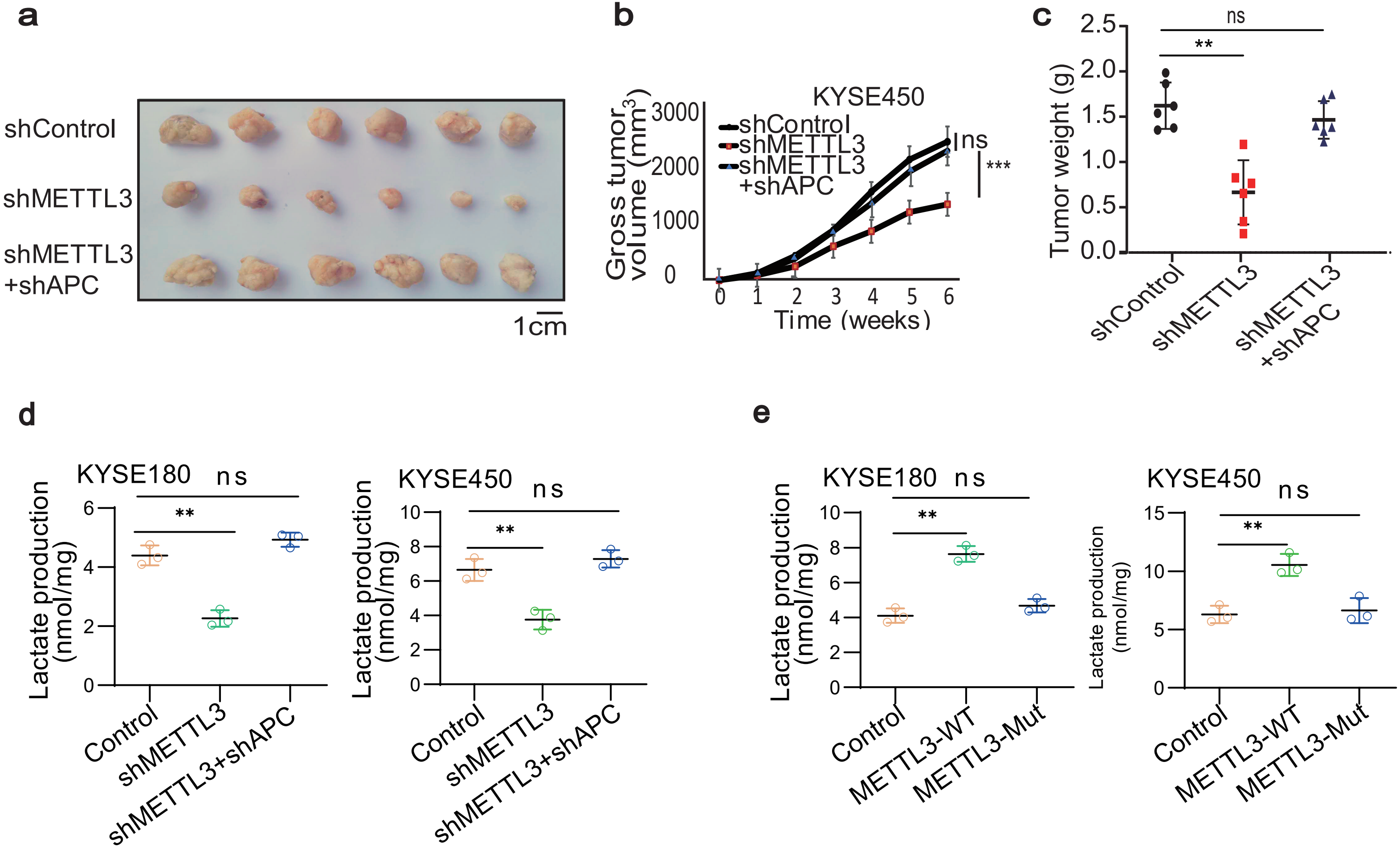

Supplementary Fig. 7 Suppression of APC expression by METTL3 promotes tumour development

(**a-c**) KYSE450 cells with or without *METTL3* shRNA expression or combined *METTL3* shRNA and *APC* shRNA expression were subcutaneously injected into the flank regions of nude mice (n = 6). Six weeks later, tumour sizes **(a)**, volumes **(b),** and weight **(c)** were measured. scale bars: 1 cm. Data represent the means ± SD of 6 mice in each group. ***p = 1.75E-08 **(b)**, **p = 0.0016 **(c)** based on two-tailed Student's t test. ns, not significant.

(**d**) KYSE180 and KYSE450 cells with or without *METTL3* shRNA expression or combined *METTL3* shRNA and *APC* shRNA expression were subcutaneously injected into the flank regions of nude mice (n = 6). Six weeks later, lactate amount in the tumour tissues were determined. Data represent the means ± SD of triplicate samples. **p = 0.0011, 0.0042 (left to right) based on two-tailed Student's t test. ns, not significant.

(**e**) KYSE180 cells with or without expressing WT METTL3 or an inactive METTL3 mutant were subcutaneously injected into the flank regions of nude mice (n = 6). Five weeks later, lactate amount in the tumour tissues were determined. Data represent the means ± SD of triplicate samples. **p = 0.0013, 0.0035 (left to right) based on two-tailed Student's t test. ns, not significant.

Source data are provided as a Source Data file.

Supplementary Table 1

Clinical characteristics of 81 ESCC patients

Characteristics		Total (cases)	Percentage（%）
all case			
Age (years)			
	≤ 60	45	55.56
	> 60	36	44.44
Sex			
	male	64	79.01
	female	17	20.99
Drinking			
	yes	51	
	not	30	62.96
Smoking			
	yes	52	
	not	29	64.2
Family History of Upper GI cancer			
	Positive	15	18.52
	Negtive	66	18.52
Grade Differenciation Grade			
	High	15	18.52
	Middle	49	60.49
	Low	17	20.99
Location			
	Upper Esophagus	8	9.88
	Middle Esophagus	34	41.98
	Lower Esophagus	39	48.15
Lymph Node Metastasis			
	Positive	33	
	Negtive	48	
TNM stagea 1			
	I/II	35	43.21
	III/IV	46	56.79

χ^2 Test was used to test the association between categorical variables

*Statistically significant

Supplementary Table 2

RT-PCR primers

Genes	5'to3'	Base number
METTL3	CCTGGGTCATTAAACTTGGAGT	22
	AAGGAGCTGTGAGCCAGTTTAT	22
METTL3	AAACTGGCTCACAGCTCCTTG	21
	TACATGATGCCATACGCTGTTG	22
METTL3	CACGTATTTGAAGACCTCTCGGA	23
	TCTTTGTGTTGACGAGGCGT	20
	CTGAGGAGCAGCTTCAGTCC	20
CTNNB1	ATTGCACGTGTGGCAAGTTC	20
	CGTCCTCGGATTCTCTGCTC	20
MYC	GCTGGTGCATTTTCGGTTGT	20
	CTGAGGAGCAGCTTCAGTCC	20
CTNNB1	ATTGCACGTGTGGCAAGTTC	20
	GGGTAATGGCAGTGTTCCCA	20
APC	GCTATCTGCGCTGCTTTTCC	20
ACTB	TGACCCAGATCATGTTTGAGA	21
	TACGGCCAGAGGCGTACAGC	20

MeRIP-PCR & RIP-PCR primers 0

Genes	5'to3'	Base number
APC	CCCGGTGATTGACAGTGTTT	20
	GGAACACTGCCATTACCCAC	20

Supplementary Table 3

Plasmids	TargetSeq
shMETTL3#1	GCTGCACTTCAGACGAATT
shMETTL3#2	GCTAAACCTGAAGAGTGATAT
shScramble	TTCTCCGAACGTGTCACGT
METTL3 rescue	GCTGCACTTCAGACGAATT mutated to ACTCCATTTTAGGCGGATC
METTL3-mut(D395A	GCTGACCCA mutated to GCTGCCCCA
shMETTL14	CCATGTACTTACAAGCCGATA
shYTHDF2#1	GGGCTGATATTGCTAGCAAGC
shYTHDF2#2	GCCAATGAGGAAAGGGCATTG
shAPC	GCAGAGAAAGTACTGGATATT
shMYC	GGAAACGACGAGAACAGTTGA
**siRNA**	**TargetSeq**
siYTHDF1	GGCTGGAGAATAACGACAACAAACC
	GCAAACATAAGAAACCATGAGTCAT
siYTHDF2	AGGAGAATATAACAGTGTT
	AGCACAGAAGTTGCAAGCA
siYTHDF3	GCAATACAGTAGATTTGAATACCTT
