# Supplementary material for: METTL3 promotes tumour development by decreasing APC expression mediated by APC mRNA N6-methyladenosine-dependent YTHDF binding
Source: Nat Commun. 2021 Jun 21;12:3803. doi: 10.1038/s41467-021-23501-5 (PMC8217513; doi:10.1038/s41467-021-23501-5)
Supplement: Supplementary file 2 — Description of Additional Supplementary Files [file 41467_2021_23501_MOESM2_ESM.docx]

Description of Additional Supplementary Files

Title: Supplementary Data 1.

Description: The genes with diminished m6A peaks identified by MeRIP-seq and the genes with regulated mRNA expression identified by RNA-seq in KYSE180 with or without METTL3 depletion.

Title: Supplementary Data 2.

Description: Supplementary source data for Figures.

Title: Supplementary Data 3.

Description: Supplementary source data for Supplementary Figures.

Title: Supplementary Data 4.

Description: Supplementary source data for Gel.
