# Supplementary material for: METTL3 promotes tumour development by decreasing APC expression mediated by APC mRNA N6-methyladenosine-dependent YTHDF binding
Source: Nat Commun. 2021 Jun 21;12:3803. doi: 10.1038/s41467-021-23501-5 (PMC8217513; doi:10.1038/s41467-021-23501-5)
Supplement: Supplementary file 4 — Reporting Summary [file 41467_2021_23501_MOESM4_ESM.pdf]

nature research

Corresponding author(s): Zhimin Lu, Jie He and Yibo Gao

Last updated by author(s): Mar 28, 2021

Reporting Summary

Nature Research wishes to improve the reproducibility of the work that we publish. This form provides structure for consistency and transparency in reporting. For further information on Nature Research policies, see our Editorial Policies and the Editorial Policy Checklist.

Statistics

For all statistical analyses, confirm that the following items are present in the figure legend, table legend, main text, or Methods section.

n/a	Confirmed	
☐	☒	The exact sample size (*n*) for each experimental group/condition, given as a discrete number and unit of measurement
☐	☒	A statement on whether measurements were taken from distinct samples or whether the same sample was measured repeatedly
☐	☒	The statistical test(s) used AND whether they are one- or two-sided<br>*Only common tests should be described solely by name; describe more complex techniques in the Methods section.*
☒	☐	A description of all covariates tested
☐	☒	A description of any assumptions or corrections, such as tests of normality and adjustment for multiple comparisons
☐	☒	A full description of the statistical parameters including central tendency (e.g. means) or other basic estimates (e.g. regression coefficient) AND variation (e.g. standard deviation) or associated estimates of uncertainty (e.g. confidence intervals)
☐	☒	For null hypothesis testing, the test statistic (e.g. *F*, *t*, *r*) with confidence intervals, effect sizes, degrees of freedom and *P* value noted<br>*Give P values as exact values whenever suitable.*
☒	☐	For Bayesian analysis, information on the choice of priors and Markov chain Monte Carlo settings
☒	☐	For hierarchical and complex designs, identification of the appropriate level for tests and full reporting of outcomes
☐	☒	Estimates of effect sizes (e.g. Cohen's *d*, Pearson's *r*), indicating how they were calculated

Our web collection on statistics for biologists contains articles on many of the points above.

Software and code

Policy information about availability of computer code

Data collection	no software was used.
Data analysis	We used the following software: Aperio ImageScope v12.3.2.7001, SPSS statistics v21, Cytoscape v3.7.2, ImageJ v2.1.4.7, GraphPad Prism 8, DREME v4.11.4, Robust Multiarray Average (RMA) v3.0.3, cutadapt software v1.9.3, Hisat2 software v2.0.4, MACS v2.2.7.1, HOMER v4.10, diffReps v1.55.3, HTSeq v0.9.1, edgeR v3.12, IGV v2.8.3 and R package MetaPlotR v0.0.3.

For manuscripts utilizing custom algorithms or software that are central to the research but not yet described in published literature, software must be made available to editors and reviewers. We strongly encourage code deposition in a community repository (e.g. GitHub). See the Nature Research guidelines for submitting code & software for further information.

Data

Policy information about availability of data

All manuscripts must include a data availability statement. This statement should provide the following information, where applicable:

- Accession codes, unique identifiers, or web links for publicly available datasets
- A list of figures that have associated raw data
- A description of any restrictions on data availability

The data of Fig. 1a, Fig. 7a and Supplementary Fig. 1a are available in a public repository from the TCGA website. The clinical records and RNAseqV2 level 3 gene level ESCC data were downloaded from TCGA [http://xena.ucsc.edu/welcome-to-ucsc-xena]. The clinical records and genome-wide gene expression profiles (Affymetrix GeneChip Human Exon 1.0 ST arrays) of a total of 119 paired ESCC datasets were downloaded from the Gene Expression Omnibus with accession number GSE53625. Gene transcription estimates for each gene were analysed using Robust Multiarray Average (RMA) software. The MeRIP-seq and mRNA-seq data have been deposited into the Gene Expression Omnibus repository under accession number GSE154555. The remaining data are available within the article, Supplementary Information or available from the authors upon request. Source data are provided with this paper.

Field-specific reporting

Please select the one below that is the best fit for your research. If you are not sure, read the appropriate sections before making your selection.

☒ Life sciences ☐ Behavioural & social sciences ☐ Ecological, evolutionary & environmental sciences

For a reference copy of the document with all sections, see nature.com/documents/nr-reporting-summary-flat.pdf

Life sciences study design

All studies must disclose on these points even when the disclosure is negative.

Sample size	In general, we used tools available at http://www.biomath.info/power/ttest.htm.
Data exclusions	No data were excluded from analysis.
Replication	Results were confirmed in at least three biological replicates for each experiment unless otherwise stated.
Randomization	No randomization was performed since this was not relevant to the study: all treatment/control experiments were performed on the same starting cell population. Controlling for covariates was unnecessary for the human samples as all comparisons were between paired tumor/normal samples collected from the same patient. We did not control for covariates in the animal experiments because all animals were the same age and sex and were purchased from the same supplier.
Blinding	No blinding was performed. This was deemed unnecessary since no clinical trials were conducted in this study and none of the analyses reported involved procedures that could be influenced by investigator bias.

Reporting for specific materials, systems and methods

We require information from authors about some types of materials, experimental systems and methods used in many studies. Here, indicate whether each material, system or method listed is relevant to your study. If you are not sure if a list item applies to your research, read the appropriate section before selecting a response.

Materials & experimental systems

n/a	Involved in the study
☐	☒ Antibodies
☐	☒ Eukaryotic cell lines
☒	☐ Palaeontology and archaeology
☐	☒ Animals and other organisms
☐	☒ Human research participants
☒	☐ Clinical data
☒	☐ Dual use research of concern

Methods

n/a	Involved in the study
☒	☐ ChIP-seq
☒	☐ Flow cytometry
☒	☐ MRI-based neuroimaging

Antibodies

Antibodies used	Rabbit monoclonal antibody recognizing β-catenin (D10A8, #8480, Lot 5) for immunoblotting was purchased from Cell Signaling Technology<br>Rabbit monoclonal antibody recognizing METTL3 (E3F2A, #86132, Lot 1) for immunoblotting was purchased from Cell Signaling Technology<br>Rabbit monoclonal antibody recognizing METTL14 (D8K8W, #51104, Lot 1) for immunoblotting was purchased from Cell Signaling Technology<br>anti-rabbit secondary antibody Boost IHC Detection Reagent (HRP, Rabbit) (#8114) for IHC was purchased from Cell Signaling Technology<br>Rabbit monoclonal antibody recognizing cyclin D1 (E3P5S, #55506, Lot 2) for immunoblotting was purchased from Cell Signaling Technology<br>Rabbit monoclonal antibody recognizing PKM2 (D78A4, #4053, Lot 2) for immunoblotting was purchased from Cell Signaling Technology<br>Mouse monoclonal antibody anti-α-Tubulin (#2144, Lot 1) for immunoblotting was purchased from Cell Signaling Technology<br>Rabbit monoclonal antibody recognizing METTL3 (EPR18810, ab195352, Lot GR3247121-4) for IHC and RIP was purchased from Abcam<br>Rabbit monoclonal antibody recognizing APC (ab15270) for immunoblotting was purchased from Abcam<br>Rabbit monoclonal antibody recognizing APC- C-terminal (ab154906, Lot GR114099-32) for immunoblotting and IHC was purchased from Abcam<br>Rabbit monoclonal antibody recognizing c-Myc (Y69, ab32072, Lot 5) for immunoblotting was purchased from Abcam<br>Rabbit polyclonal antibody recognizing YTHDF1 (17479-1-AP) for RIP and immunoblotting was purchased from Proteintech<br>Rabbit polyclonal antibody recognizing YTHDF2 (24744-1-AP) for RIP and immunoblotting was purchased from Proteintech

April 2020

Rabbit polyclonal antibody recognizing YTHDF3 (25537-1-AP) for RIP and immunoblotting was purchased from Proteintech
Rabbit polyclonal antibody normal rabbit IgG (# 2729) for immunoprecipitation was purchased from Cell Signaling Technology
Rabbit polyclonal antibody anti-m6A antibody (202 003) for MeRIP was purchased from Synaptic Systems

Validation

In general, we relied on data provided by the manufacturers for validation as well as references in publications.
Rabbit monoclonal antibody recognizing β-catenin (D10A8) for WB, IP, IHC, IF, F, CHIP in human, mouse, rat, monkey
Rabbit monoclonal antibody recognizing METTL3 (E3F2A) for WB, IP, CHIP in human, mouse, rat, monkey
Rabbit monoclonal antibody recognizing METTL14 (D8K8W) for WB in human, mouse, rat, monkey
anti-rabbit secondary antibody (HRP, Rabbit) (#8114, Lot 21) for IHC in human, mouse, house
Rabbit monoclonal antibody recognizing cyclin D1 (E3P5S) for WB, IP, IHC, IF, F in human, mouse, rat
Rabbit monoclonal antibody recognizing PKM2 (D78A4) for WB, IP, IHC, IF, F in human, mouse, rat, monkey
Mouse monoclonal antibody anti-α-Tubulin (#2144, Lot 1) for WB, IHC, IF, F in human, mouse, rat, monkey
Rabbit monoclonal antibody recognizing METTL3 (ab195352) for IP, Flow Cyt, ICC/IF, IHC-P, WB in mouse, rat, human
Rabbit monoclonal antibody recognizing APC (ab15270) for HC-P in human
Rabbit monoclonal antibody recognizing APC- C-terminal (ab154906) for ICC/IF, IHC-P, WB in human
Rabbit monoclonal antibody recognizing c-Myc (Y69, ab32072) for WB, IP, IHC, IF, F in human, mouse, rat,
Rabbit polyclonal antibody recognizing YTHDF1 (17479-1-AP, Lot 00044548) for WB, IP, RIP, IHC, IF, ELISA in human, mouse, rat
Rabbit polyclonal antibody recognizing YTHDF2 (24744-1-AP, Lot 00053880) for WB, IP, RIP, IHC, IF, ELISA in human, mouse, rat
Rabbit polyclonal antibody recognizing YTHDF3 (25537-1-AP, Lot 00049687) for WB, IP, RIP, IHC, IF, ELISA in human, mouse, rat
Rabbit polyclonal antibody normal rabbit IgG (# 2729, Lot 10) for IP, CHIP in human, mouse, rat
Rabbit polyclonal antibody anti-m6A antibody (202 003) for ICC, IP, WB, IHC-P, ELISA in human, rat, mouse, eukaryotes, prokaryotes

Eukaryotic cell lines

Policy information about cell lines

Cell line source(s)

TE1, TE10, KYSE30, KYSE70, KYSE140, KYSE150, KYSE180, KYSE410, KYSE450, HeLa, HEK293T and Het-1a cell lines were obtained from ATCC.

Authentication

TE1, TE10, KYSE30, KYSE70, KYSE140, KYSE150, KYSE180, KYSE410, KYSE450 HeLa, HEK293T and Het-1a cell lines were authenticated with STR profiling.

Mycoplasma contamination

Cells were confirmed to be free of mycoplasma.

Commonly misidentified lines
(See ICLAC register)

No commonly misidentified cell lines were used.

Animals and other organisms

Policy information about studies involving animals; ARRIVE guidelines recommended for reporting animal research

Laboratory animals

These female BALB/c nude mice (4-5 weeks) purchased from Beijing HFK bioscience. All mice were successfully entrained by a light-dark cycle. Temperatures of 18-23°C with 40-60% humidity.

Wild animals

The study did not involve wild animals.

Field-collected samples

The study did not involve filed-collected samples.

Ethics oversight

This study was approved by the Ethics Committee of the National Cancer Center/Cancer Hospital, Chinese Academy of Medical Sciences, and Peking Union Medical College.

Note that full information on the approval of the study protocol must also be provided in the manuscript.

Human research participants

Policy information about studies involving human research participants

Population characteristics

Information about the patient sex, age, and tumor characteristics are given in Supplementary Table 2. There were 64 males and 17 females, aged 39-82 years, with esophageal squamous cell carcinoma of different stages and degrees. All of patients underwent radical resection. Controlling for covariates was not necessary as the analyses compared tumor and adjacent normal tissues taken from the same patient.

Recruitment

Participants were recruited from the pool of ESCC patients at Thoracic Surgery of the Cancer Hospital, Chinese Academy of Medical Sciences. The sample should be representative of the populations served by these institutions and reflect the general biological characteristics of ESCC.

Ethics oversight

This study was approved by the Ethics Committee of the National Cancer Center/Cancer Hospital, Chinese. Academy of Medical Sciences, and Peking Union Medical College and Peking Union Medical College.

Note that full information on the approval of the study protocol must also be provided in the manuscript.
